# The dystroglycan receptor maintains glioma stem cells in the vascular niche

**DOI:** 10.1007/s00401-019-02069-x

**Published:** 2019-08-28

**Authors:** Bryan W. Day, Justin D. Lathia, Zara C. Bruce, Rochelle C. J. D’Souza, Ulrich Baumgartner, Kathleen S. Ensbey, Yi Chieh Lim, Brett W. Stringer, Seçkin Akgül, Carolin Offenhäuser, Yuchen Li, Paul R. Jamieson, Fiona M. Smith, Courtney L. R. Jurd, Thomas Robertson, Po-Ling Inglis, Zarnie Lwin, Rosalind L. Jeffree, Terrance G. Johns, Krishna P. L. Bhat, Jeremy N. Rich, Kevin P. Campbell, Andrew W. Boyd

**Affiliations:** 1 Department of Cell and Molecular Biology, Sid Faithfull Brain Cancer Laboratory, QIMR Berghofer Medical Research Institute, Brisbane, QLD 4006 Australia; 2 Faculty of Health, Queensland University of Technology, Brisbane, 4059 Australia; 3 Faculty of Medicine, The University of Queensland, Brisbane, 4072 Australia; 4 Cleveland Clinic, Lerner College of Medicine, Case Western Reserve University, Cleveland, OH 44195 USA; 5 Royal Brisbane and Women’s Hospital, Brisbane, QLD 4006 Australia; 6 Telethon Kids Institute, Perth, WA 6009 Australia; 7 Department of Translational Molecular Pathology, The University of Texas, MD Anderson Cancer Center, Houston, TX 77030 USA; 8 Medicine Department, University of California, La Jolla, San Diego, CA 92093-0021 USA; 9 Department of Molecular Physiology and Biophysics, Roy J. and Lucille A. Carver College of Medicine, Howard Hughes Medical Institute, University of Iowa, Iowa City, IA 52242 USA; 10 Department of Neurology, Roy J. and Lucille A. Carver College of Medicine, Howard Hughes Medical Institute, University of Iowa, Iowa City, IA 52242 USA

**Keywords:** Glioblastoma (GBM), Dystroglycan (DG), MES-like GBM, EphA3, Integrin-α6, Glioma stem cell (GSC) commitment, Perivascular niche, MAPK signalling

## Abstract

Glioblastomas (GBMs) are malignant central nervous system (CNS) neoplasms with a very poor prognosis. They display cellular hierarchies containing self-renewing tumourigenic glioma stem cells (GSCs) in a complex heterogeneous microenvironment. One proposed GSC niche is the extracellular matrix (ECM)-rich perivascular bed of the tumour. Here, we report that the ECM binding dystroglycan (DG) receptor is expressed and functionally glycosylated on GSCs residing in the perivascular niche. Glycosylated αDG is highly expressed and functional on the most aggressive mesenchymal-like (MES-like) GBM tumour compartment. Furthermore, we found that DG acts to maintain an MES-like state via tight control of MAPK activation. Antibody-based blockade of αDG induces robust ERK-mediated differentiation leading to reduced GSC potential. DG was shown to be required for tumour initiation in MES-like GBM, with constitutive loss significantly delaying or preventing tumourigenic potential in-vivo. These findings reveal a central role of the DG receptor, not only as a structural element, but also as a critical factor promoting MES-like GBM and the maintenance of GSCs residing in the perivascular niche.

**Electronic supplementary material:**

The online version of this article (10.1007/s00401-019-02069-x) contains supplementary material, which is available to authorized users.

## Introduction

Glioma stem cells (GSCs) have been shown to reside in both the necrotic and perivascular regions of the tumour, where glioblastoma (GBM) cells adopt a more stem cell-like phenotype [3, 9, 11]. Recently, single-cell RNA sequencing studies have identified four GBM cellular states (MES-like, NPC-like, OPC-like and AC-like) that exist in varying degrees within single tumours [46]. This seminal study highlights the diversity and heterogeneity present within these aggressive tumours and provides a framework for better understanding the genetic diversity, plasticity and influence of the tumour microenvironment (TME) in GBM.

Extracellular matrix (ECM) proteins and basement membrane (BM) formation are key structural components required to support the TME in GBM [25]. The dystrophin–glycoprotein complex (DGC) is a large transmembrane oligomeric complex of sarcolemmal proteins and glycoproteins. A central component of the DGC is the dystroglycan (DG) receptor. DG is an ECM binding receptor best known for its function in bridging the ECM and the cytoskeleton in skeletal myocytes [30]. The DG receptor comprises two non-covalently linked α and β subunits. αDG, the extracellular component, is highly glycosylated. Laminin binds a glycan (carbohydrate) structure attached to αDG and this interaction is critical in regulating ECM attachment. βDG, the membrane spanning component, primarily regulates dystrophin attachment to F-actin cables within the cell [18, 19, 32]. Dysregulation of αDG glycosylation or DG loss leads to various forms of muscular dystrophy, the most well-known being Duchenne muscular dystrophy (DMD) [17, 20]. DG has also been shown to be critical for brain development with receptor dysregulation leading to a number of structural and functional brain defects [43, 44]. In comparison to skeletal muscle function, less is known about the role of the DGC in adult brain. DG has been linked to blood–brain barrier (BBB) function and is present on the perivascular end feet of astrocytes, where it mediates cell adhesion to vascular ECM proteins [29, 41, 47, 48]. DG also has an established role in regulating oligodendrocyte differentiation and function [2, 12, 22, 35]. More recently, Colognato and colleagues revealed that DG is present in the subventricular zone (SVZ) and regulates neural stem and progenitor cell proliferation via suppression of Notch signalling in the postnatal brain [39].

Eph receptor tyrosine kinases and integrin family receptors have been well described as having functional roles in GSC niche formation and maintenance. Eph receptors were first described during development and have been shown to be functionally over expressed in a wide variety of human cancers [7, 31, 51]. Our previous work and the findings of others have shown that a particular family member, EphA3, is significantly elevated in MES-like GBM, is most highly expressed on GSCs, particularly in perivascular regions, and is significantly elevated in recurrent disease [13, 24, 54]. Moreover, EphA3 has been shown to be over expressed and functional on mesenchymal stromal cells in the TME of human cancers [58]. Recently, we have shown that EphA3 can be effectively targeted in GBM animal models using pay-loaded antibody therapeutic strategies [50]. Integrin receptors, particularly integrin α6 is also expressed on GSCs in the perivascular niche and functions to promote tumourigenesis [11, 34].

The role of αDG in GSC commitment and its contribution to GBM cellular states is yet to be elucidated. Here, we identify a central role of the αDG receptor in promoting a GBM MES-like state and maintenance of GSCs in the vascular niche via tight regulation of ERK signalling. Our findings indicate that αDG is functionally glycosylated and cooperates with EphA3 and integrin α6 receptors to mediate an MES-like state and further acts to anchor GSCs residing in perivascular tumour regions.

## Materials and methods

### Experimental model and subject details

#### Human samples

Human brain cancer specimens were collected from the Royal Brisbane and Women’s Hospital (RBWH) under Human Ethics approved project HREC/17/QRBW/577: Novel Therapies for Brain Cancer. Specimens were collected immediately following resection and processed for analysis at QIMR Berghofer laboratories.

#### Primary cell cultures

We have developed a characterised GBM patient-derived cell line resource, data is publicly available from Q-Cell https://www.qimrberghofer.edu.au/q-cell/ [14, 57] GBM lines are maintained as glioma neural stem cell (GNS) cultures [52] or as neurosphere cultures using StemPro NSC SFM (Invitrogen) as per manufacturer’s guidelines. U251-MG GBM cells were obtained from the ATCC and cultured as neurospheres as described above.

#### Animal studies

All mice experiments were performed according to the National Health and Medical Research Council (2013) Australian code for the care and use of animals for scientific purposes, under experimental protocols approved by the QIMR Berghofer Animal Ethics Committee.

#### Animal strain

For intracranial (orthotopic) xenograft studies, five week-old female NOD/SCID (NOD.CB17-Prkdc scid/Arc, animals were sourced from the Animal Resources Centre (ARC), Canning Vale Western Australia.

#### Orthotopic engraftment

Primary lines derived from human GBM specimens were transduced with lentiviral vectors expressing multiple combined *DAG1* shRNA targeting sequences, or expressing a non-targeting control shRNA. Cells were counted (1.6 × 10^4^ cells for WK1 GNS—4 animals per group; 1.5 × 10^5^ cells for JK2 GNS—7 animals per group) and engrafted intracranially into the right striatum (0.8 mm lateral of the midline, 1.6 mm caudal to the bregma, at a depth of 3 mm) using a small animal stereotactic device. Mice were given analgesia (Meloxicam (Ilium) 5 mg/kg, delivered subcutaneously) 30 min prior to surgery and again the following day. Mice were monitored daily for signs of illness or tumour burden, as per our ethical guidelines, animal monitoring criteria and scoring. At endpoint, animals were euthanised by cervical dislocation. Brains were collected and fixed in 10% neutral - buffered formalin for 24 h, transferred to 70% ethanol, then subsequently embedded in paraffin. Sections were cut (4 μm) and stained for H&E according to common methods, using a Leica ST5010 Autostainer XL and Leica CV5030 Fully Automated Glass Coverslipper (both Leica Biosystems).

## Method details

### RNA isolation and real-time PCR

Total cellular RNA was isolated from tissue or cell lines using TRIzol reagent (Thermo Scientific). RNA was DNase treated using RQ1 RNase-Free DNase (Promega), then first strand cDNA was synthesised using random hexamers (Random Primer 6, New England BioLabs) SuperScript III Reverse Transcriptase (Thermo Scientific), and dNTPs (Promega). Real-time PCR was performed using a Viia 7 Real-Time PCR System and SYBR-Green PCR Master Mix (both Thermo Scientific). Results were normalised to β-actin (*ACTB*), with expression represented as copy number per 1000 β-actin. Sequences for gene-specific primer sets were as in Table 1:

**Table 1:** Gene-specific primer sets

#	Primer	Forward 5′–3′	Reverse 5′–3′
1	*ACTB* (β-actin)	CACACTGTGCCCATCTACGA	GTGGTGGTGAAGCTGTAGCC
2	*DAG1*	AGGCAGATCCATGCTACACC	AGGATCCCTGACTGGAGGAG
3	*ITGA6A*	CCACATATCACAAGGCTGAG	CACTGTCATCGTACCTAGAG
4	*ITGA6B*	ATTCTCGCTGGGATCTTGATG	GATCCTTACAGCATGGTATCGG
5	*CDH1*	GGCTGATACTGACCCCACAG	CGTACATGTCAGCCAGCTTC
6	*CDH2*	CCTTGATCTGATGTTTGTGG	CCTGGTCTTCTTCTCCA
7	*SNAI2*	TGTTGCAGTGAGGGCAAGAA	GACCCTGGTTGCTTCAAGGA
8	*SNAI1*	ACCACTATGCCGCGCTCTT	GGTCGTAGGGCTGCTGGAA
9	*TWIST1*	GGAGTCCGCAGTCTTACGAG	TCTGGAGGACCTGGTAGAGG
10	*VEGFA*	AGGGCAGAATCATCACGAAGT	AGGGTCTCGATTGGATGGCA
11	*BMI1*	TGCTGGAGAACTGGAAAGTG	GATGAGGAGACTGCACTGGA
12	*VIM*	AGATGGCCCTTGACATTGAG	CCAGAGGGAGTGAATCCAGA
13	*EPHA3*	GATGTTGGTGCTTGTGTTGC	GTGTCTGGAAACATAGCCAGATT
14	*EPHA2*	GGGACCTGATGCAGAACATC	AGTTGGTGCGGAGCCAGT
15	*FUT4* (CD15)	TACGATTTGTGCCCCGGCGC	GATAGACCGCGGGGTTGCGG
16	*PROM1* (CD133)	GCCACCGCTCTAGATACTGC	TCGTACACGTCCTCCGAATC
17	*ITGA6* (CD49f)	TCATGGATCTGCAAATGGAA	AGGGAACCAACAGCAACATC
18	*SOX2*	GCGAACCATCTCTGTGGTCT	GGAAAGTTGGGATCGAACAA
19	*TUBB3* (βIII-tubulin)	AACGAGGCCTCTTCTCACAA	GGCCTGAAGAGATGTCCAAA
20	*OLIG2*	AACAGTTTGGGTTATTTGGG	GGAGGAGTTTACCATTTGTG
21	*ESRP1*	GGGAGTTCGCCACAGATATTC	AGCCATAAATGCTCTGTCCG

All primers were against human sequences

### Cellular fractionation and western blotting

Total cellular protein was isolated using 8 M Urea with phosphatase and protease inhibitors. Membrane-and-cytoplasmic protein fractions was isolated using a Mem-PER Plus Membrane Protein Extraction kit (Life Technologies). Equal amounts of protein were loaded, separated by SDS/PAGE and immunoblotted using standard methods. For Western blotting, we used αDG (Millipore IIH6C4, 1:1000), EphA2 (CST 6997, 1:1,000) and EphA3 (Invitrogen, 373,200, 1:1,000), ERK1/2 (CST 4695, 1:1,000), and p-ERK1/2 (CST 4370, 1:1,000). β-Actin was used as a loading control (CST 3700, 1:2,000).

### Immunohistochemistry

Immunohistochemistry experiments were performed using OCT-embedded frozen specimens from patient brain tumours. 6 μm sections were cut, fixed in a solution of 2:1 acetone:ethanol for 5 min, incubated in 3% hydrogen peroxide (Chem-supply) for 10 min to block endogenous peroxidase activity, and incubated in Background Sniper (Biocare Medical) for 30 min to block non-specific binding, then incubated with the following primary antibodies at 4 °C overnight: Vimentin, clone V9 (DAKO M0725, 1:400), 1F7 (in-house anti-EphA2, 1:75), IIIA4 (in-house anti-EphA3, 1:50), α-dystroglycan/LARGE-glycan, clone VIA4-1 (Merck Millipore 05-298, 1:75), β-dystroglycan (B-4) (Santa Cruz Biotechnology sc-165997, 1:100), CD31 (M-20) (Santa Cruz Biotechnology sc-1506 1:50), GFAP, clone GA-5 (Biocare Medical CM 065, 1:500), CDW49f, clone 4F10 (Merck Millipore BCL458, 1:25), Ki-67 (Abcam ab66155, 1:300), Caspase-3 (Biocare Medical CP 229, 1:80), Phospho-p44/42 MAPK (Erk1/2) (Thr202/Tyr204) (Cell Signalling Technology 4370 s, 1:400), following by incubation with a MACH1 or MACH2 HRP Polymer Detection (Biocare Medical), according to the manufacturers instruction, together with the 3,3′-Diaminobenzidine (DAB) Chromogen Kit (Biocare Medical), for detection. Sections, where then counterstained (Mayer’s Haematoxylin), dehydrated, cleared and coverslipped, using a Leica ST5010 Autostainer XL and Leica CV5030 Fully Automated Glass Coverslipper (both Leica Biosystems).

### Flow cytometry

Expression was analysed using primary antibodies with the following specificities: α-Dystroglycan (EMD Millipore, IIH6C4, 1:100), EphA2 (in-house, 1F7, 1:100), EphA3 (in-house, IIIA4, 1:100), CD133 (Miltenyi Biotec, AC133, 1:20), CD49f (Abcam, GoH3, 1:200), CD29 (Abcam, EP16895, 1:100) and CD15 (Abcam, SP159, 1:200). IgG1 and IgM antibodies (Abcam, 1:100) were used to indicate background staining. Cells were washed twice in PBS and blocked for 20 min in PBS supplemented with 1% BSA (Sigma). After blocking, cells were incubated with primary antibodies for 20 min at room temperature (RT). Washed cells were then incubated for 20 min at 4 °C in the dark with secondary antibodies Anti-Mouse IgM Alexa Fluor 488 (Abcam, 1:2000), Anti-Rabbit Alexa Fluor 647 (Abcam, 1:2000) and/or Anti-Rat Alexa Fluor 647 (Abcam, 1:2000). Before analysis cells were washed in blocking solution to remove any unbound antibody conjugates. Between 20,000 and 50,000 total events were examined using an LSRFortessa (BD) with data analysed using FACSDiva (BD) software. Live cells were gated using propidium iodide (Sigma, 50 μg/ml) staining.

### Amnis analysis

The flow cytometry procedure was repeated for GBM cell lines, as described above. Samples were then run on an ImageStream^x^. Briefly, 5,000 events were collected for each sample and single color controls used to create a compensation matrix to correct for spectral overlap. All data were then analyzed using IDEA software (Amnis Corporation, Seattle, WA, USA).

### Immunofluorescence

#### Perivascular tissue staining

Dual IF experiments were performed using OCT-embedded frozen specimens from patient GBM tumours cut, fixed, incubated in hydrogen peroxide and Background Sniper as described under IHC. Sections were incubated with α-dystroglycan antibody (Abcam ab106110, 1:50, 4 °C overnight) and goat anti-mouse AF488 (1:300 RT for 1 h) followed by purified IgG mouse Fab fragments (1:4000 RT for 10 min) and rodent block Fab fragment 5 (1:50 RT for 1 h) to block any free mouse antibody. The second staining was performed with CD31/PECAM, Clone -JC70A (Dako M0823, 1:30, RT 2 h), goat anti-mouse AF647 (1:300 RT 1 h) and coverslipped with DAKO fluorescent mounting media and visualized using a Zeiss confocal microscope.

#### Differentiation marker staining

Single cells were suspended in StemPro NSC SFM (Invitrogen) and seeded in 24-well plates containing sterile coverslips. Cells were then treated with α-Dystroglycan inhibiting antibody (EMD Millipore, IIH6C4, 1:100) or an IgM control antibody (Mo1, in-house, 1:100) for 24 h. After treatment, adherent and tumoursphere cultures were fixed in 4% PFA for 15 min at RT. Cells were then blocked and expression of extracellular proteins α-Dystroglycan, EphA3 and EphA2 were analysed as previously described. Expression of differentiation markers βIII-tubulin (Promega G712A, 1:100), GFAP (DAKO 20,334, 1:100) and myelin basic protein (Sigma M3821, 1:100) were analysed by permeabilising fixed cultures in 0.25% Triton X-100 for 10 min at 4 °C.

#### Nuclear ERK translocation

Single cells were seeded on coverslips in a 24-well plate and growth factor starved overnight. Cells were then treated with α-Dystroglycan inhibiting antibody (EMD Millipore, IIH6C4, 1:100) or an IgM control (Mo1, in-house, 1:100) for 24 h prior to fixing and permeabilisation, as described. A control well treated with FBS (Gibco, 2%) was also included. Expression of ERK translocation was observed using the antibody phosphorylated-ERK (Cell Signalling, D13.14.4E, 1:200).

#### DAG1 knockdown validation

Single cells were seeded on coverslips coated in Matrigel (Corning, 1:200) and α-Dystroglycan expression was analysed as previously described.

### α-Dystroglycan antibody blockade

Single cells were suspended in StemPro NSC SFM (Invitrogen) without growth factors and seeded in 6-well plates (5 × 10^4^ per well) in triplicate and treated with α-Dystroglycan inhibiting antibody (EMD Millipore, IIH6 C4, 1:100, 50 µg/ml) or an IgM control (Mo1, in-house, 1:100, 50 µg/ml). After 7 days, adherent and neurosphere cultures were collected and dissociated using accutase, proliferation was calculated by direct cell counting using a haemocytometer.

### IncuCyte

Single GBM cells (1 × 10^3^) were plated onto individual wells (96-well format) for 16 h prior to experimentation. Cells were treated with α-Dystroglycan inhibiting antibody (EMD Millipore, IIH6C4, 1:100, 50 µg/ml) or an IgM control (Mo1, 1:100, 50 µg/ml). The Incucyte system (Essen Instruments) was employed to quantitate cell adhesion, as a surrogate read out for differentiation, in real time.

### Apoptosis

Apoptosis and viability was assessed using the ApoTox-Glo Triplex assay (Promega) as described [4]. Cleaved caspase3/7 activity and cell viability was assessed 48 h post IIH6 treatment, 7.5 mg/mL cisplatin treatment was used as a positive control. Results are shown as caspase3/7 activity normalised to cell viability relative to IgM control (*n* = 3).

### Cell culture and transduction

Target cells were plated on Matrigel-coated (Corning, 1:200) plates in StemPro NSC SFM (Invitrogen) at 50% confluency 24 h prior to viral infection. The next day, plating media was removed and polybrene (Santa Cruz, 5 μg/ml) added, followed by the addition of the Lentiviral Particles α/β-Dystroglycan shRNA (Santa Cruz, sc-43488-V, 20 μl/ml) and control shRNA (Santa Cruz, sc-108080, 20 μl/ml). 24 h following transduction, fresh media was added and cells underwent puromycin (Gibco, 0.3 μl/ml) selection.

### MAPK activation studies

#### CD49f KD and mAb-blocking studies

CD49f was down regulated using previous validated integrin α6 shRNA sequences compared to a control shRNA [34]. Target cells were plated at 50% confluency 24 h prior to transfection. The next day, plating media was removed and cells were transfected using a FuGENE^®^HD:DNA ratio of 3.0:1 (Promega). FACSAria IIIu (BD) was used to select positive clones based on GFP expression. Expression KD was confirmed via flow cytometry using CD49f (Abcam, GoH3, 1:200). Dissociated cells were seeded in 24-well plates and treated with α-Dystroglycan inhibiting antibody (EMD Millipore, IIH6C4, 1:100) or an IgM control (Mo1, in-house, 1:100).

Single cells were seeded in 6-well plates and growth factor starved overnight. Pre-blocked cells were then treated with CD49f (Invitrogen, eBioGoH3, 10 μg/ml) 24 h prior to treatment with α-Dystroglycan inhibiting antibody (EMD Millipore, IIH6C4, 1:100) or an IgM control (Mo1, in-house, 1:100). After 3 h incubation cells were collected and pERK (Cell Signalling, D13.14.4E, 1:2000) and total ERK (Cell Signalling, 137F5, 1:1000) expression was analysed via western blot.

#### EGFR inhibitor studies

Single cells were seeded in 24-well plates and growth factor starved overnight. Cells were then treated with Erlotinib (2 μM) or Gefitinib (2 μM) 24 h prior to treatment with α-Dystroglycan inhibiting antibody (EMD Millipore, IIH6C4, 1:100) or an IgM control (Mo1, in-house, 1:100).

### Quantification and statistical analysis

In-vivo experiments—sample size or replicate number (designated as “*n*”) for each experiment are indicated in the figure. Survival plots were generated using Graphpad software. A Log-rank (Mantel-Cox) test was used to determine significance between experimental groups. *P-*values are as indicated, *p ≤ 0.05 was considered to be statistically significant. In-vitro experiments—a Student’s t-test determined the probability of difference; *p* < 0.05 was considered significant; all statistical tests were 2-sided. The correlation coefficient was determined using a Spearman rank.

## Results

### Elevated dystroglycan correlates with glioma patient outcome and αDG is abundantly glycosylated in GBM

**Fig. 1:**
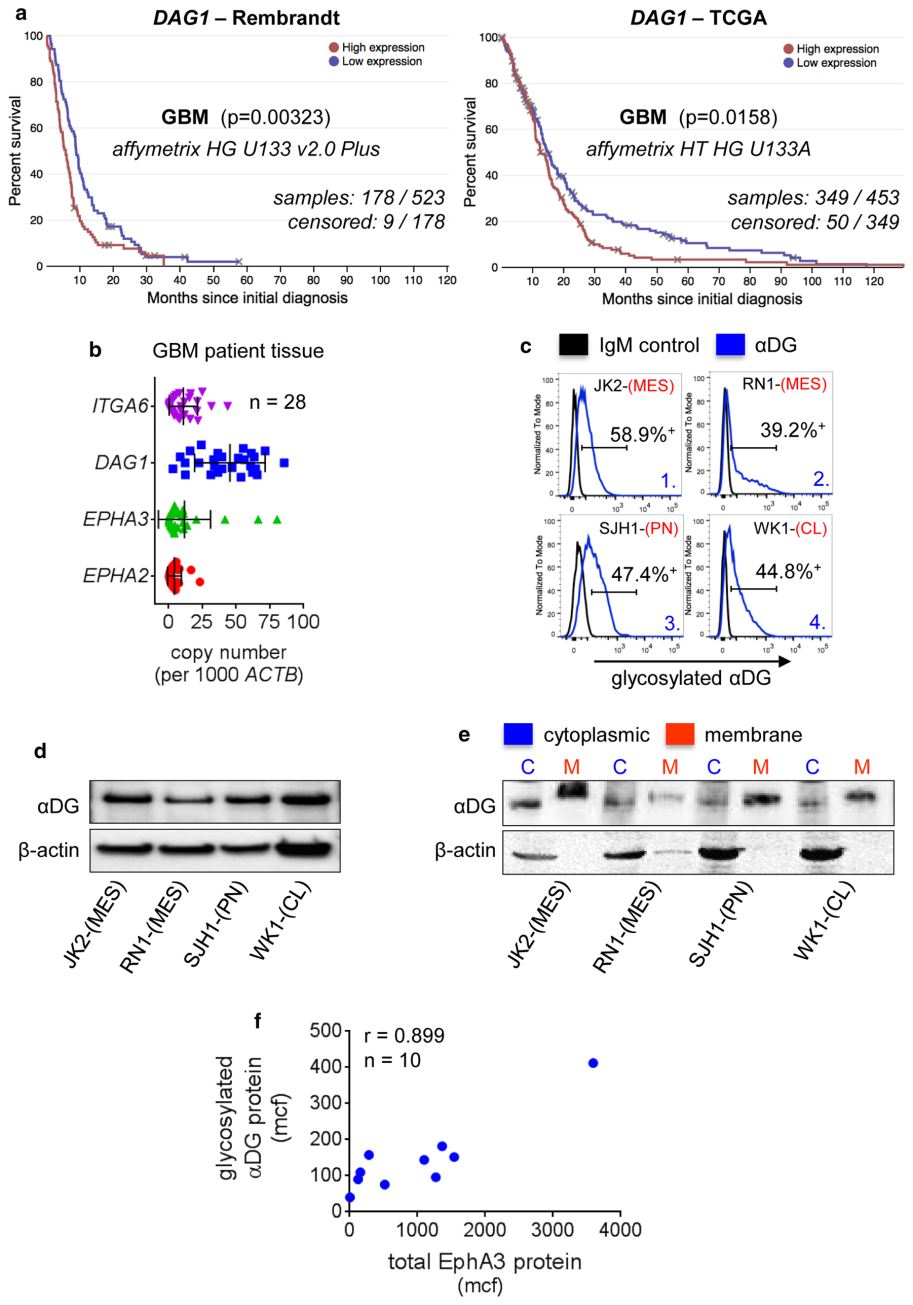
Elevated Dystroglycan Correlates with GBM Patient Outcome and αDG is Abundantly Glycosylated in GBM. **a***DAG1* expression was correlated with GBM patient survival using the Rembrandt (*n* = 523) and TCGA (*n* = 453) databases. **b** QPCR analysis of *DAG1, ITGA6, EPHA2* and *EPHA3* mRNA expression in GBM tissue specimens (*n* = 28). **c** Flow cytometric analysis for αDG glycosylation (IIH6 mAb) was performed on primary GBM cell lines grown as serum-free GNS cultures, compared to isotype control. See also Online Research 1d for full analysis. **d** αDG glycosylation was assessed by western blot in four primary GBM cell lines. **e** αDG glycosylation was assessed by western blot following cell fractionation to compare cytoplasmic versus membrane localisation. β-actin was used as a loading control. **f** Flow cytometric analysis was performed for αDG glycosylation (IIH6 mAb) and EphA3 (IIIA4 mAb) in 10 early passage primary GBM cultures, mean channel fluorescence (mcf) was used to determine the correlation coefficient between EphA3 and glycosylated αDG (*r* = 0.899). GBM subtypes: *MES* mesenchymal, *PN* proneural, *CL* classical

αDG and βDG are transcribed from the Dystrophin-Associated Glycoprotein 1 (*DAG1*) gene. DG is translated as a single pre-pro-polypeptide. The receptor is autocatalytically cleaved into alpha and beta chains that remain non-covalently bound. The mucin-like domain of αDG becomes heavily glycosylated en-route to the surface membrane. Dystroglycan binds proteins of the extracellular matrix such as laminin by virtue of matriglycan (xylose and glucuronic acid polysaccharide). Receptor glycosylation is essential for proper function, without which αDG is unable to bind laminin [19, 42]. To determine if *DAG1* was important in the context of brain cancer we interrogated both the Rembrandt and TCGA databases to correlate *DAG1* gene expression with survival. In the context of GBM specifically and also glioma, patient tumours with elevated *DAG1* led to a significantly shorter survival time (Fig. 1a and Online Resource 1a). The Rembrandt database was also used to assess *DAG1* gene expression in GBM as well as other forms of malignant brain cancer versus normal brain tissue. This revealed that *DAG1* tended to correlate with tumour grade, as expression was highest in GBM compared to oligodendroglioma and astrocytoma cases, while all tumour types were elevated above normal brain tissue (Online Resource 1b). *DAG1* expression in GBM was further stratified into molecular subtype [8, 59, 60]. *DAG1* expression was highest in classical (CL) subtype GBM while approximately equivalent in other subtypes, mesenchymal (MES), proneural (PN) (Online Resource 1c). To assess the relative mRNA levels of *DAG1* in GBM we performed QPCR on 28 GBM tumour specimens from our in-house tumour bank. We compared expression to other receptors (*EPHA2*, *EPHA3* and *ITGA6*) previously identified as having a role in GSC maintenance. *DAG1* levels were equivalent or higher to these receptors in all cases assessed (Fig. 1b).

Importantly, receptor function correlates closely with glycosylation status rather than gene expression. To determine the level of αDG glycosylation we used a monoclonal antibody (mAb) (IIH6), previously developed by Campbell and colleagues, specific to glycan moieties on αDG with known receptor blocking function [20]. We have developed a GBM patient-derived cell line resource (Q-Cell) [14, 57] in which GBM lines are maintained as glioma neural stem cell (GNS) cultures [52]. This approach promotes a more de-differentiated stem cell-like phenotype in culture. We assessed a panel (*n* = 20) of subtype classified (*n* = 12) and unclassified (*n* = 8) early passage GBM cultures generated in-house. Flow cytometric analysis revealed the majority of the lines expressed high levels of glycosylated αDG irrespective of GBM molecular subtype (Fig. 1c and Online Resource 1d). A panel of four IDH1 WT GBM lines were selected for further analysis, JK2-(MES), RN1-(MES), SJH1-(PN) and WK1-(CL), αDG protein expression was confirmed by western blot (WB) (Fig. 1d). We next performed cell fractionation studies followed by WB on the panel of GBM lines, data indicated the majority of the protein was present in the cell membrane (Fig. 1e).

A previous report in breast cancer had shown that EphA3 could regulate αDG [63]. Given these findings we assessed the co-expression of glycosylated αDG and EphA3 protein levels by flow cytometry. Mean channel fluorescence (mcf) analysis revealed a high correlation (*r* = 0.899) between glycosylated αDG and EphA3 in the primary GBM GNS lines tested (*n* = 10) (Fig. 1f).

### Glycosylated αDG is expressed in the vascular niche and discretely on mesenchymal-like glioma tissue

**Fig. 2:**
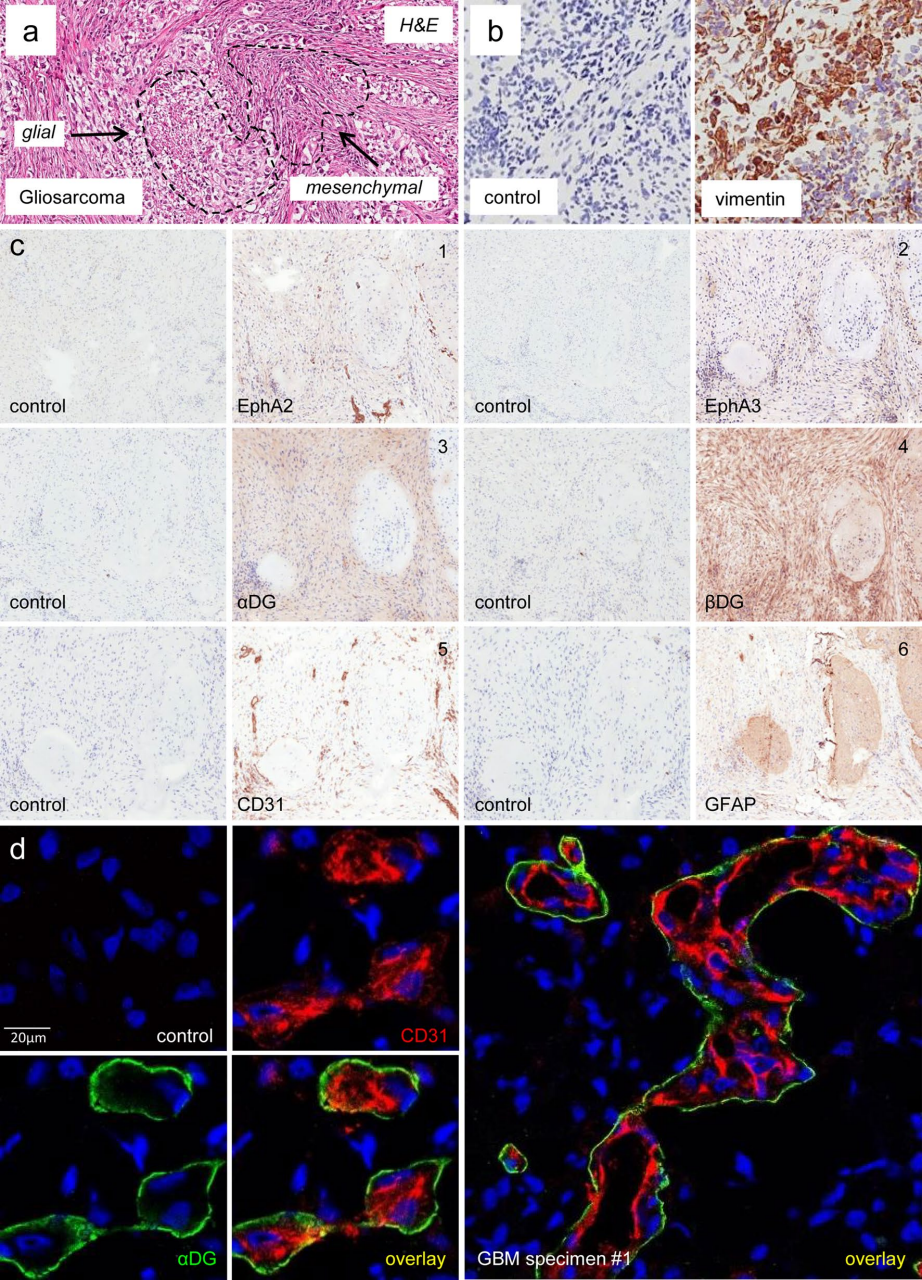
Glycosylated αDG is expressed in the vascular niche and discretely on Mesenchymal-like glioma tissue. **a** H&E section of a Gliosarcoma (GS) patient specimen showing characteristic biphasic gliomatous (glial-like) and sarcomatous (mesenchymal-like) tumour elements. **b** IHC analysis of a GS specimen to identify the mesenchymal-like, vimentin^+^, tumour element. **c** IHC analysis of sequential (#1–6) GS tissue sections was performed to assess expression patterns of EphA2, EphA3, αDG, βDG, CD31 and GFAP. **d** IF dual staining of a GBM specimen showing localisation of αDG expression (IIH6—green) surrounding (CD31^+^—red) tumour blood vessels, nuclei DAPI—blue). See also Online Resource 2 for additional IHC and IF analysis in GBM tissue specimens

To examine the spatial localisation and tumour specificity of glycosylated αDG (hereafter stated as αDG for simplicity), we first examined a variant of GBM, gliosarcoma (GS), which undergoes a partial process of de-differentiation with secondary loss of the glial differentiation marker GFAP and acquisition of mesenchymal characteristics, a process similar to epithelial–mesenchymal transition (EMT) [45]. GS is characterised histologically by a biphasic pattern of gliomatous glial-like (GFAP^+^) and sarcomatous mesenchymal-like (MES-like) (vimentin^+^) tumour elements (morphology shown in Fig. 2a) [27, 45]. Common genetic alterations are found between GBM and GS, and both have a poor prognosis. A known clinical feature of GS is that the mesenchymal tumour compartment is highly migratory and infiltrates ECM protein-rich brain regions such as the leptomeninges [6]. As previously shown by others, the mesenchymal elements stain positive for vimentin [27]. We performed immunohistochemistry (IHC) for vimentin, and found a clear separation between glial-rich and predominantly mesenchymal-rich regions (Fig. 2b). We next performed IHC on sequential GS tumour tissue sections to determine the localisation of αDG, βDG, EphA2 and EphA3. A representative example shows that αDG and EphA3 are expressed predominantly in regions, where mesenchymal tumour elements are present and expression is low or absent on the more differentiated GFAP^+^ glial tumour element (Fig. 2c). This discrete pattern was not evident for EphA2 and βDG which showed comparable expression in both glial and mesenchymal tumour elements. The observed biphasic tumour tissue pattern in GS also appeared, although less dramatically discrete, in GBM specimens (Online Resource 2a). These regions were smaller and less prominent than in GS tumours, and also showed heterogeneous expression of GFAP, vimentin, EphA2, EphA3 αDG, CD49f and CD31, indicating that this tumour tissue patterning might also occur in GBM (Online Resource 2b). In addition, immunofluorescent (IF) staining revealed in GBM patient specimens (n = 3) that αDG staining was strongest in perivascular regions immediately surrounding tumour blood vessels (CD31^+^) (Fig. 2d and Online Resource 2c).

### αDG interacts with EphA2 and EphA3 receptors and is expressed on GSCs

**Fig. 3:**
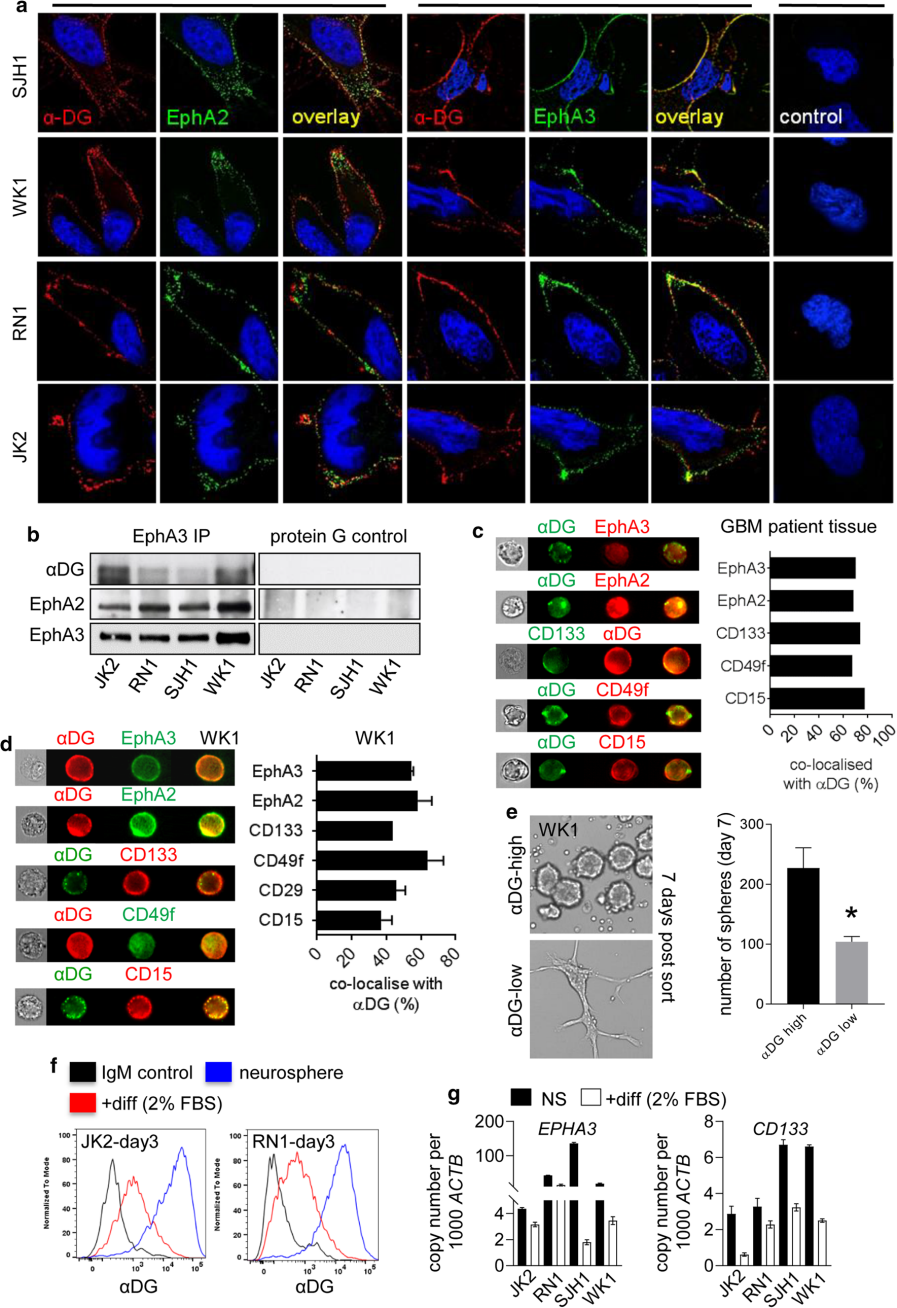
αDG Interacts with EphA2 and EphA3 Receptors and is Expressed on GSCs. **a** IF staining was performed to compare membrane localisation of glycosylated αDG (IIH6 mAb—red), compared to either EphA2 (1F7 mAb—green) or EphA3 (IIIA4 mAb—green), in four primary GBM cultures. **b** Immunoprecipitation (IP) for EphA3 was performed from 1 mg of lysate from four early passage primary GBM cultures to compare membrane association of EphA2, EphA3 and glycosylated αDG. Protein G only was used as a control. **c** Amnis flow cytometric analysis was performed on a dissociated GBM patient tissue specimen (*n* = 1) to assess membrane localisation of glycosylated αDG, with known GSC markers (CD15, CD133, CD49f, EphA2 and EphA3). **d** Amnis analysis and flow cytometry was performed on primary GBM cultures to assess membrane localisation of glycosylated αDG, with known GSC markers. **e** αDG^high^ versus αDG^low^ populations were isolated from WK1 cells using FACS and cell morphology and sphere number assessed 7 days post-sort (*p < 0.05). GBM neuropshere differentiation was induced using 2% FBS, 3 days post-differentiation glycosylated αDG expression was assessed by flow cytometry and GSC and diff marker expression assessed by QPCR and compared to undifferentiated cells, see Online Resource 3 for complete analysis. All data presented as the mean ± SD of three independent experiments

Given the overlapping expression profile of EphA3 and αDG in GS and GBM tissue we wanted to assess the membrane localisation and potential interaction between these receptors. IF analysis showed a significant overlap in the membrane of EphA2, EphA3 and αDG in the four primary GBM cell lines tested (Fig. 3a). To assess if these proteins could be complexed together we performed immunoprecipitation (IP) of EphA3 in these lines. Results show co-immunoprecipitation of EphA3 with EphA2 and αDG (Fig. 3b). To further confirm an association between these receptors we performed shRNA mediated EphA3 knockdown (KD) in SJH1 and JK2 cell lines. Following EphA3 KD a commensurate drop in αDG levels was observed (Online Resource 3a). These data suggest that αDG and EphA3 are likely associated in the cell membrane.

We next used Amnis image stream technology, a flow cytometric approach which captures single-cell multi-colour fluorescent images. This approach was used to assess cell-surface co-expression of αDG with known GSC markers. Initially, this was performed on dissociated GBM patient tumour tissue (n = 1) obtained directly at time of surgery. Results show strong overlapping expression of αDG with EphA2, EphA3, CD49f (integrin α6), CD133 and CD15 (Fig. 3c). We repeated this analysis using both Amnis and standard flow cytometry using the panel of GBM cell lines (*n* = 4). Results demonstrated a similar pattern of co-expression of αDG especially with EphA2, EphA3 and CD49f, this was less pronounced for CD15, CD29 and CD133 (Fig. 3d and Online Resource 3b,c). To assess stemness characteristics in the αDG high versus low expressing population we sorted WK1 GBM cells using FACS and performed a neurosphere assay. Results show that neurosphere formation was significantly elevated in the αDG^high^ compared to the αDG^low^ population. In addition, we observed cell changes in the αDG^low^ population; low expressing cells that failed to form neurospheres displayed an attached differentiated cell morphology (Fig. 3e). Post αDG^high^ versus αDG^low^ sort we recombined the two cell populations and assessed αDG expression overtime. Interestingly, 14 days post-sort both low and high expressing populations were maintained, suggesting that these two unique populations can co-exist in culture for extended periods (Online Resource 3d). αDG expression and stemness characteristics should be lost following GSC differentiation. To test this we induced differentiation in our neurosphere cultures and 72 h post-differentiation assessed αDG expression by flow cytometry and GSC marker expression by QPCR. Results show a marked reduction in αDG protein expression following differentiation (Fig. 3f), this was accompanied by significant gene downregulation of stemness markers and upregulation of the differentiation markers *GFAP*, βIII-tubulin (*TUBB3*) and *OLIG2* (Fig. 3g and Online Resource 3e).

### αDG blockade induces GSC differentiation

**Fig. 4:**
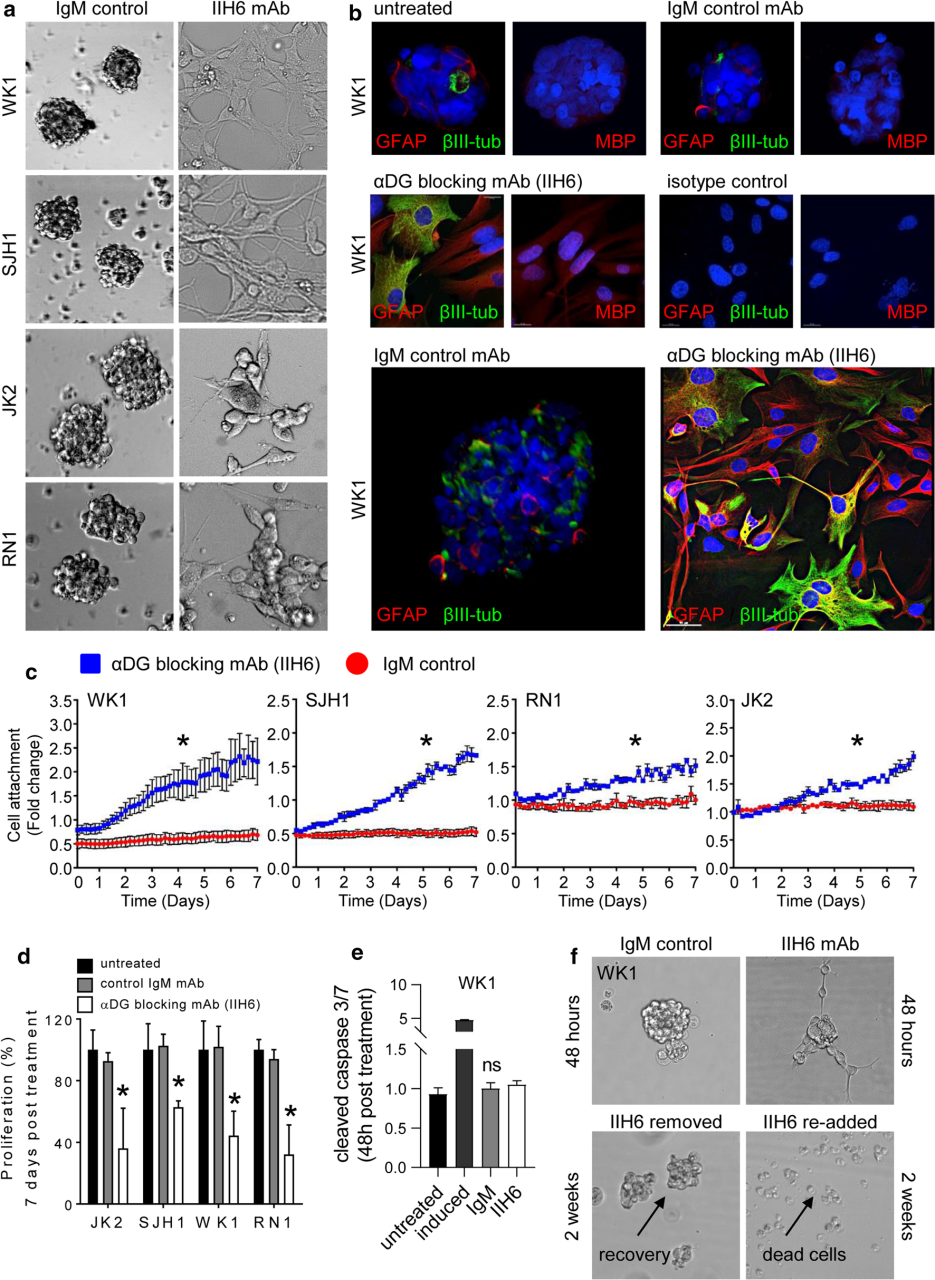
αDG Blockade Induces GSC Differentiation. a Four primary GBM neurosphere cultures treated with the αDG blocking mAb (IIH6, 50 µg/ml) and an equivalent IgM control (50 µg/ml). Bright field images 24 h post-treatment showing loss of neurosphere formation and cell adherence. **b** IF staining was performed 48 h post IIH6 mAb treatment for the differentiation markers (GFAP-red, βIII-tubulin—green, myelin basic protein (MBP)—red and the nuclear counter-stain DAPI-blue). WK1 data shown see also Online Resource 4b for complete analysis. **c** IncuCyte analysis was performed to quantitate cell adhesion in real time following IIH6 treatment-blue compared to an equivalent IgM control-red for 7 days (*p < 0.05). **d** Cell proliferation was assessed by direct cell counting using a haemocytometer 7 days post IIH6 treatment compared to IgM control and untreated cells (*p < 0.01). **e** ApoTox-Glo Triplex assay was used to assess caspase3/7 activity and cell viability 48 h post IIH6 treatment. WK1 data shown see also Online Resource 4c for complete analysis. f Neurosphere formation was assessed in WK1 cells following IIH6 withdrawal or re-addition following 7 days initial treatment. Bright field images at 2 weeks showing neurosphere re-formation following IIH6 withdrawal and cell death following IIH6 rechallenge. Data presented as the mean ± SEM of three independent experiments

To assess the contribution of αDG glycosylation to the progression of GBM and maintenance of a GSC phenotype, we employed an αDG mAb (IIH6) which specifically binds and blocks the ability of glycan moieties on αDG to bind laminin [20]. Following incubation of neurospheres with the IIH6 antibody, we observed a robust and rapid loss of sphere formation compared to cultures incubated with an isotype control mAb in which sphere integrity was maintained. The response was reliably observed in all primary cultures tested (Fig. 4a). The observed differentiation response was dose dependent, while no response was observed with three independent IgM control antibodies (Online Resource 4a). Neurosphere loss is suggestive of differentiation and loss of proliferative ability. Differentiation was confirmed by acquisition of neuronal (βIII-tubulin) and glial (GFAP) lineage markers (Fig. 4b and Online Resource 4b). This differentiation response was quantitated using IncuCyte technology in real time and showed pronounced cell-attachment following IIH6 treatment (Fig. 4c). The observed differentiation response was accompanied with a significant reduction in proliferation (*p* < 0.01) (Fig. 4d). We observed negligible cell death induction in the short term (48 h) following antibody treatment (Fig. 4e and Online Resource 4c). Interestingly, the differentiation effects were reversible if antibody was removed within seven days of treatment. If additional IIH6 mAb was added at seven days, the majority of rechallenged tumour cells showed pronounced cell morphology changes and ultimately underwent cell death within two weeks (Fig. 4f). These data highlight the ability of GBM cells to recover during early differentiation but also susceptibility to prolonged terminal differentiation induced by the IIH6 antibody.

### αDG controls ERK signalling to regulate GSCs and promote an MES-like GBM state

**Fig. 5:**
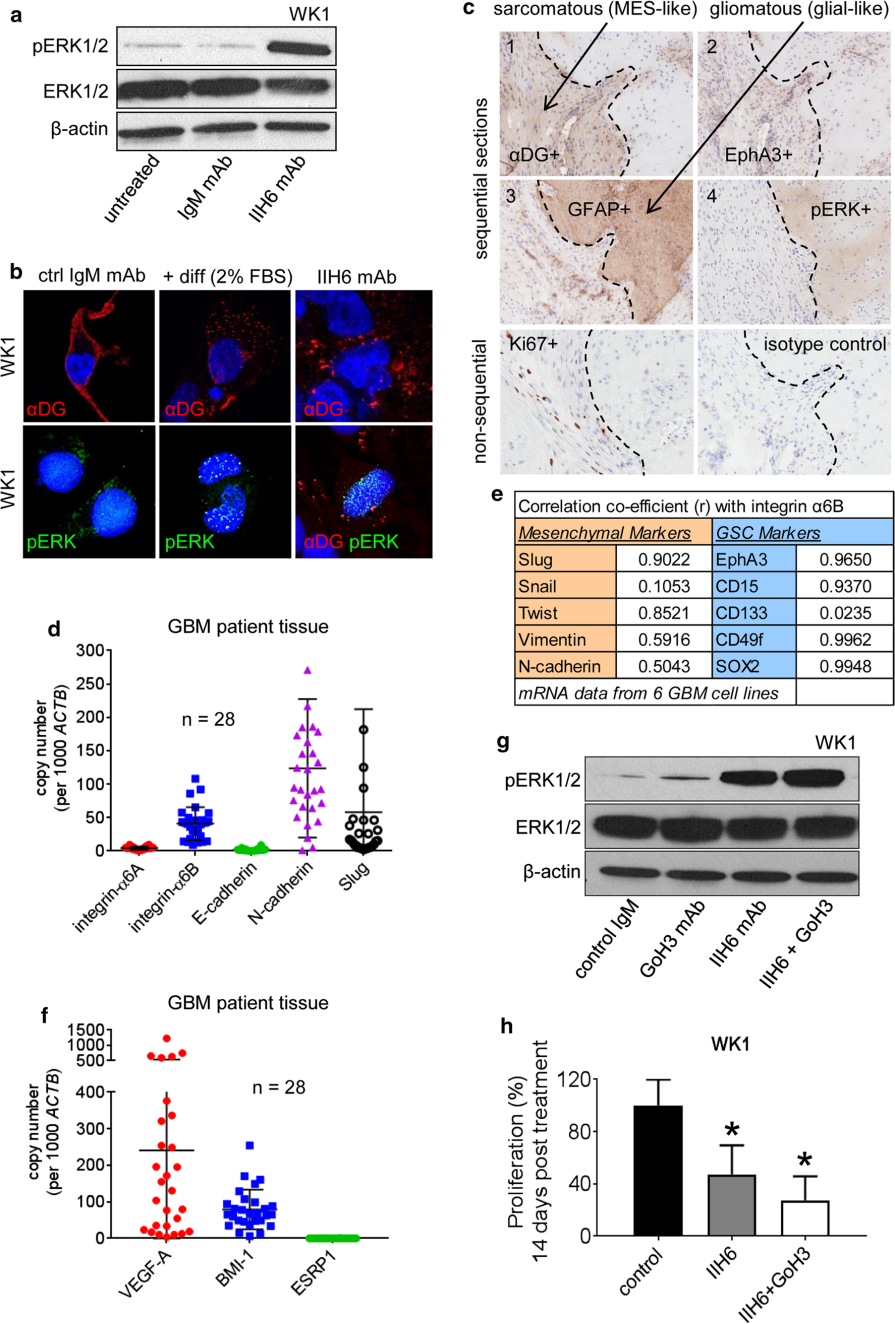
αDG Controls ERK Signalling to Regulate GSCs and Promote an MES-like GBM State. a ERK1/2 phosphorylation (pERK1/2) was assessed by western blot in WK1 GBM neurospheres following IIH6 (50 µg/ml) treatment for 24 h, compared an equivalent IgM control mAb (50 µg/ml) and untreated cells. β-Actin was used as a loading control. b IF staining was performed 24 h post IIH6 mAb treatment for pERK1/2—green and αDG—red to assess nuclear versus cytoplasmic localisation, 2% FBS was used as positive differentiation control. **c** IHC analysis of sequential (#1–4) GS tissue sections was performed to assess discrete expression patterns of αDG, EphA3, GFAP, pERK and Ki67 (non-sequential). **d** QPCR analysis of integrin α6A, integrin α6B, E-cadherin, N-cadherin and Slug mRNA expression in GBM specimens (*n* = 28). **e** Correlation coefficient analysis of mRNA expression of the mesenchymal markers (Slug, Snail, Twist, Vimentin and N-cadherin) and the GSC markers (EphA3, CD15, CD133, CD49f and SOX2) in 6 primary early passage GBM GNS cultures. f QPCR analysis of VEGF-A, BMI-1 and ESRP1 mRNA expression in GBM specimens (*n* = 28). g ERK1/2 phosphorylation (pERK1/2) was assessed by western blot in WK1 GBM neurospheres following GoH3 (10 µg/ml) or IIH6 (50 µg/ml) either alone or in combination compared an equivalent control mAb combination IgG2a (10 µg/ml)/IgM(50 µg/ml), β-actin was used as a loading control. h Cell proliferation was assessed by direct cell counts using a haemocytometer 7 days post IIH6 treatment alone or combined with GoH3. Data presented as the mean ± SD of three independent experiments, *p < 0.05

Sustained ERK activation, as distinct from transient signalling, induces translocation of ERKs to the nucleus resulting in pro-differentiation transcriptional changes in neuronal cells [38]. Based upon our previous work and also the studies of others, it appears this mechanism is also observed in a number of cancers including GBM [13, 16]. We, therefore, assessed ERK activation following IIH6 treatment and found that pERK activation was sustained 24 h post-treatment (Fig. 5a). Analysis of pERK cellular localisation, following IIH6 treatment, showed that activated ERK was predominantly localised the nucleus. The observed response was equivalent to a positive differentiation control (2% FBS), while in control-treated cells, the majority of pERK was retained in the cytoplasm (Fig. 5b). To examine if this same mechanism was operative in patient tissue and not an artefact of culture we assessed sequential (#1–4) GS tumour sections by IHC for αDG, EphA3, GFAP, pERK and Ki67. Results confirmed co-expression of αDG and EphA3 in the mesenchymal fraction which was discrete from the more differentiated GFAP^+^ glial fraction. pERK IHC staining revealed clear co-expression with the GFAP^+^ glial-predominant tumour regions which also showed less proliferation as shown by very low level Ki67 staining (Fig. 5c). This data confirmed that sustained ERK activation occurs in high-grade glioma tissue and is restricted to the more differentiated, GFAP^+^, less mitotically active tumour regions.

A study in breast cancer found that tumour cell populations differ in expression of integrin α6 cytoplasmic domain splice variants; α6A is expressed on epithelial tumour cells while α6B on mesenchymal tumour cells [26]. Data show that α6B is the critical variant driving EMT and cancer stem cell function and promotes tumour initiation. Laminin-integrin interactions can activate ERK but only in cells expressing predominantly α6A with an intact cytoplasmic tail, not α6B [61]. While DG when bound to laminin, can suppress integrin a6-mediated ERK activation [23]. Integrin α6 has previously been demonstrated to be expressed and functional in the GSC niche and is typically active in the process of niche formation and basement membrane attachment [11, 34]. We assessed α6A and α6B mRNA expression via QPCR in 28 GBM specimens. Results show that α6B and the mesenchymal markers N-cadherin and Slug are predominantly expressed, while α6A and the epithelial marker E-cadherin are essentially absent (Fig. 5d). We also investigated the relative mRNA levels of α6A and α6B in 6 primary GBM GNS cell lines and found an average 12.1 fold increase in α6B compared to α6A. This correlated with an average 210 fold increase in the mesenchymal marker N-cadherin compared to the E-cadherin (Online Resource 5a). In addition, α6B correlated highly with mesenchymal and GSC markers in the primary GNS lines tested (*n* = 6) (Fig. 5e). The oncogene BMI-1 is a stem cell factor and polycomb group family member that functions as a repressor of the splicing factor ESRP-1. Mercurio and colleagues show, in breast cancer, that α6B is positively controlled by autocrine VEGF-A signalling that culminates in transcriptional repression of the RNA-splicing factor ESRP-1 [26]. We, therefore, assessed BMI-1, VEGF-A and ESRP-1 mRNA levels by QPCR in GNS cell lines (*n* = 6) (data not shown) and GBM tissue (*n* = 28), this revealed high levels of BMI-1 and VEGF-A, while ESRP-1 levels were undetected (Fig. 5f). This suggests that a similar mechanism to that observed in breast cancer could be operative in GBM. To determine if integrin α6 was responsible for driving sustained ERK activation following IIH6 treatment we performed an integrin α6 shRNA mediated KD compared to control shRNA. These cells were then treated with the IIH6 blocking mAb, KD of integrin α6 did not reverse the differentiation response. A similar response was observed when cells were pre-treated with the integrin α6 blocking mAb GoH3 (Online Resource 5b, c). The GoH3 blocking antibody had little effect when used as a single agent on multiple GNS cell lines and did not appear to induce a differentiation response (data not shown). GoH3 treatment alone appeared to induce very low level ERK activation (Fig. 5g). This was expected given the majority of integrin α6 present is the α6B splice variant with a truncated intracellular tail with reduced ERK signalling capacity. When IIH6 was combined with GoH3 we observed a somewhat more rapid differentiation response, this coincided with an increased level of sustained ERK activation than IIH6 alone 24 h post-treatment (Fig. 5g), and a greater reduction in cell proliferation (Fig. 5h). Indicating that the sustained ERK activation, observed following IIH6 treatment, was not driven through integrin α6.

As described above, EphA3 is co-localised with dystroglycan and integrin α6. Following EphA3 IP we observed co-immunoprecipitation of EGFR in GBM cells, suggesting that EGFR is also present as a part of this complex and may be involved in regulating ERK activation (Online Resource 5d). To test if EGFR was responsible for driving sustained ERK activation following antibody treatment, neurospheres were pre-treated with either Gefitinib (2 µM) or Erlotinib (2 µM) prior to IIH6 treatment. EGFR inhibition did not prevent the induction of differentiation (Online Resource 5e). Taken together, this data suggests that αDG when bound to laminin, forms a complex with EphA2, EphA3, integrin α6B, EGFR and likely other receptors to mediate a de-differentiated GSC mesenchymal-like phenotype through tight regulation of ERK signalling.

### *DAG1* down-regulation delays or prevents GBM formation in-vivo

**Fig. 6:**
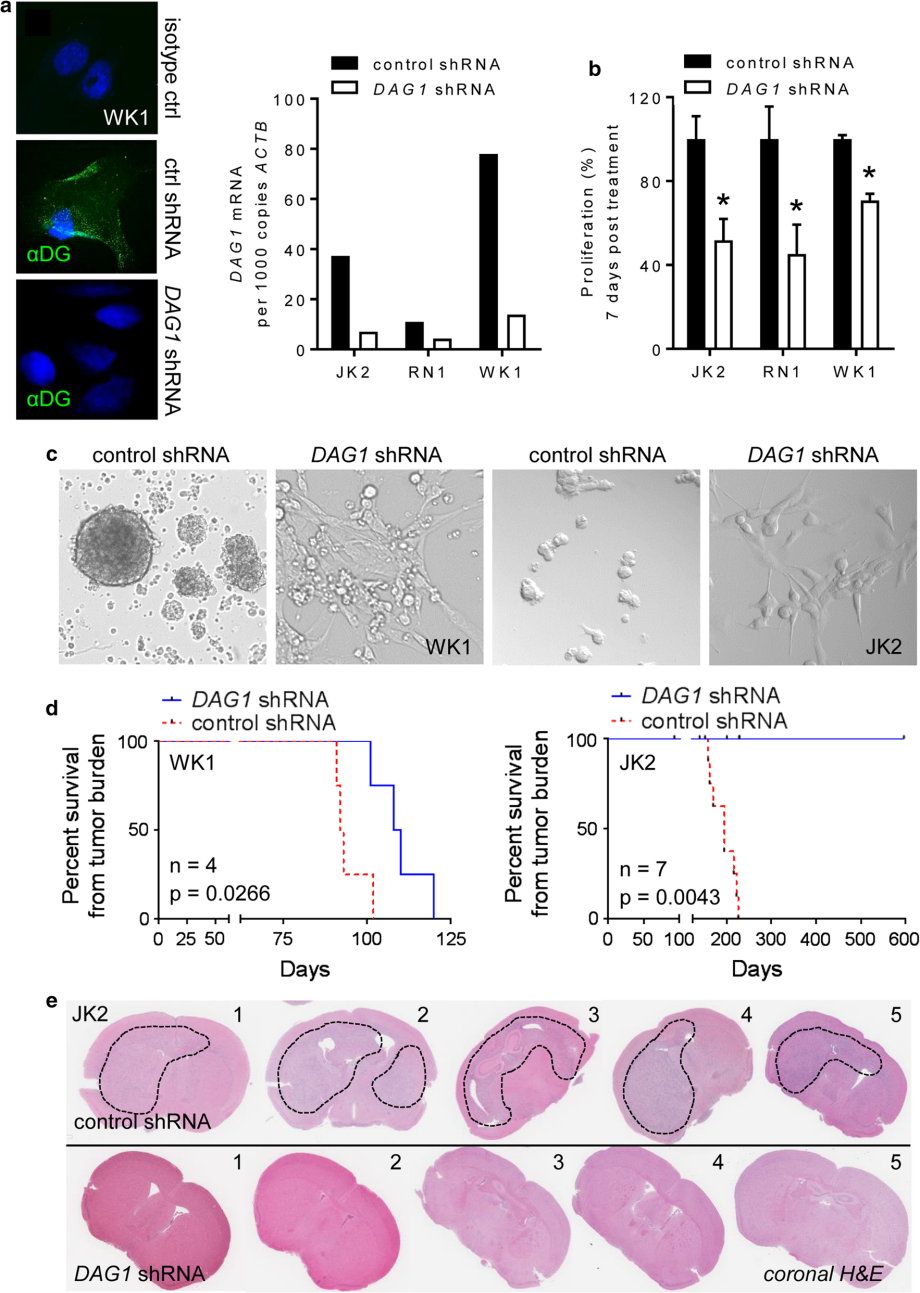
*DAG1* down regulation delays or prevents GBM formation in-vivo*.***a** IF staining and QPCR was used to assess *DAG1* mRNA levels following shRNA mediated KD in primary GBM cell lines. **b** Cell proliferation was assessed by direct cell counts using a haemocytometer following stable *DAG1* KD. Data presented as the mean ± SD of three independent experiments, *p < 0.05. **c** Bright field images of WK1 and JK2 neurospheres 2 week post-*DAG1* shRNA KD compared to control shRNA transfected cells. See also Online Resource 6a for SJH1 images. **d** Kaplan Meier analysis showing a significant *p* < 0.05 increase in overall survival following orthotopic intracranial engraftment of 1 × 10^5^*DAG1* shRNA versus control shRNA cells into the right striatum of NOD-SCID mice. WK1 *n* = 4 animals per arm, JK2 *n* = 7 animals per arm. **e** Representative H&E coronal sections from *DAG1* shRNA versus control shRNA engrafted animal following euthanasia from either illness or tumour burden. See Online Resource 6d for WK1 H&E images

To further define DG function in GBM we performed lentiviral *DAG1* shRNA mediated KD, using a combination of five target-specific 19–25 nucleotide sequences against *DAG1,* four GBM neurospheres lines were tested. Following effective constitutive KD in JK2, RN1 and WK1 cells (Fig. 6a), we observed a significant (*p* < 0.05) reduction in proliferation (Fig. 6b) and neurosphere loss typical of differentiating GBM cells (Fig. 6c). In the case of SJH1, we observed a florid response to *DAG1* KD; all cells underwent cell death within 7–14 days following KD (Online Resource 6a). Given this rapid response, SJH1 cells were excluded from further analysis. In-vitro* DAG1* KD also reduced expression of EphA2, EphA3 and CD49f (integrin α6) while markedly increasing the expression of the glial differentiation marker GFAP, indicating KD was inducing glial lineage differentiation (data shown for JK2) (Online Resource 6b). To explore if *DAG1* KD reduced the tumourigenic potential of GBM cells in-vivo we performed orthotopic engraftment of control shRNA versus *DAG1* KD cells into the right striatum of NOD/SCID mice. Animals were euthanised when signs of illness or disease burden were observed. The two highest *DAG1* expressing lines (WK1-(CL), JK2-(MES)) were selected. Kaplan Meier survival analysis for WK1 showed *DAG1* KD significantly increased overall survival *p* = 0.0266 (*n* = 4), median survival for control was 94 days versus *DAG1* KD 109 days (Fig. 6d). qPCR analysis of post-mortem tumour tissue confirmed a significant (*p* < 0.01) reduction in *DAG1* mRNA levels compared to control shRNA engrafted animals (Online Resource 6c). Positive tumour formation was confirmed by haematoxylin and eosin (H&E) staining in all control and *DAG1* KD engrafted animals (Online Resource 6d). Kaplan Meier survival analysis for JK2 showed *DAG1* KD significantly increased overall survival *p* = 0.0043 (*n* = 7), with no animals reaching survival endpoint, whereas median survival for control group was 195 days (Fig. 6d). All *DAG1* KD animals developed spontaneous thymic lymphoma, which is a common occurrence in this strain, and were euthanised as per our ethical guidelines [53]. Positive tumour formation was confirmed by H&E staining in all control shRNA engrafted animals, while no clinical signs of intracranial tumour formation were observed in *DAG1* KD animals or intracranial tumour detected by H&E staining post-mortem (Fig. 6e).

## Discussion

While decades of research have greatly increased our understanding of GBM, this knowledge is yet to translate into meaningful survival benefit for patients. We are now only starting to fully appreciate the true complexity and functional cellular states that reside within these aggressive tumours [46]. This plasticity and complex interaction with the TME is largely responsible for the modest clinical progress achieved to-date. Solid tumours, such as GBM, also harbour populations of cancer cells with stem cell-like abilities. These GSCs have the potential for self-renewal, tumour initiation and recurrence and often reside in necrotic and perivascular regions [28]. A better understanding of GSC biology and the functional elements that drive these dynamic cellular states in GBM will be required before meaningful clinical advances are likely to be made.

Dystroglycan biology and function in the brain is relatively understudied and less is known in the context of GBM. Concordant with our findings, Petrucci and colleagues found that transmembrane βDG was highly expressed on the majority of brain tumour tissue. They reported reduced αDG expression in two serum grown immortalised GBM cell lines (U87-MG and A172-MG) and showed positive expression in one EphA3^+^ GBM cell line (U251-MG), which we subsequently confirmed [10]. By flow cytometric analysis, we found significant αDG expression in 17/20 early passage primary GBM samples grown as serum-free GNS cultures. There appeared to be no correlation of expression with molecular subtype, this was expected as GNS cultures were grown on a basement membrane of laminin or matrigel which tends to promote a more MES-like de-differentiated phenotype irrespective of molecular subtype [5, 59, 62]. Our IHC and IF expression analysis demonstrated strong αDG expression around brain tumour blood vessels in patient specimens. This perivascular expression pattern confirms those of an earlier study [49]. We also detected abundant αDG expression in vimentin^+^, EphA3^+^, GFAP^low^ MES-like tumour regions. The number of Ki67 positive cells was elevated in αDG^+^/EphA3^+^/vimentin^+^ tumour regions compared to other regions, suggesting this tumour compartment was more mitotically active. Furthermore, we noted distinct morphological differences, αDG^+^/EphA3^+^/vimentin^+^ regions had an elongated, mesenchymal morphology, while negative regions had a more rounded, immotile appearance. While evident in both GBM and the GS tumours, this observation was most marked in GS and is consistent with disease biology, where the vimentin^+^ MES-like (sarcomatous) tumour cells are highly motile and migrate to BM-rich regions in the brain such as the leptomeninges [36, 37, 45]. GS is an uncommon variant of GBM and, despite exhibiting distinct sarcomatous and gliomatous tumour morphologies, are monoclonal tumours with GBM-associated genetic alterations [6]. While GBM are not epithelial tumours, the mesenchymal differentiation observed in GS tumours has been reported to occur through an EMT-like process [45]. In GBM we observed a similar, less prominent, pattern of MES-like αDG^+^/vimentin^+^ tumour tissue often detected in large tumour sections, indicating that GBM may also undergo a similar EMT-like process. Recently seminal studies from Suva and colleagues have described four states that exist within single GBM tumours to varying degrees. These states (MES-like, NPC-like, OPC-like and AC-like) are all driven by unique elements within the tumour and display unique properties [46]. They show that the MES-like NF1-driven regions express vimentin highly and interact frequently with the TME and immune infiltrate. Similar to our findings, they show that MES-like tissue contained considerable subsets of proliferating cells, reflecting the aggressive nature of the MES-like tumour compartment. Taken together, our data suggests that αDG, along with EphA3, are critical components of this MES-like tumour state, promoting functional interaction with BM proteins likely within the TME and perivascular niche.

We also observed a strong co-expression of αDG with other known GSC markers. This is not surprising given the recently identified roles of DG in regulating neural stem and progenitor cell commitment in the postnatal SVZ [39]. We found disruption of the strong connection of DG to laminin, either by αDG mAb blockade or *DAG1* downregulation, led to pronounced loss of stem cell-like characteristics and induction of differentiation. Interestingly, the *in-vitro* effects of αDG mAb blockade were reversible within one week of treatment, while prolonged treatment resulted in tumour cell death suggestive of irreversible, terminal differentiation. *In-vivo DAG1* KD studies were conducted using two models, WK1 (CL) and JK2 (MES), in the later mesenchymal model no tumours were detected in all *DAG1* KD animals including a single animal which survived for 598 days post engraftment. Suggesting a strong reliance on αDG to tumour progression in this mesenchymal subtype model.

The strong link we found between αDG and EphA3 further confirmed our previous findings connecting EphA3 to an MES-like GSC phenotype [13]. A recent report by Singh and colleagues have shown that EphA3 is significantly elevated in recurrent GBM and cooperates with EphA2 to promote tumourigenesis [54]. Based on these findings, αDG may also be enriched in recurrent GBM. Sustained ERK activation induces translocation of ERKs to the nucleus resulting in pro-differentiation transcriptional changes in normal neuronal cells and some forms of cancer [16, 38]. In our previous work, we found EphA3 KD in GBM induced sustained ERK activation which induced differentiation and reduced cell growth and tumourigenicity [13]. This appeared true for αDG, antibody-mediated receptor blockade also led to sustained ERK activation and translocation of ERKs to the nucleus followed by GSC differentiation and ultimately cell death. We also observed this phenomenon in patient tissue, where more differentiated, GFAP^+^ Ki67^low^ vimentin^low^, tumour regions stained positive for phosphorylated ERK1/2, highlighting an active process during disease progression.

**Fig. 7:**
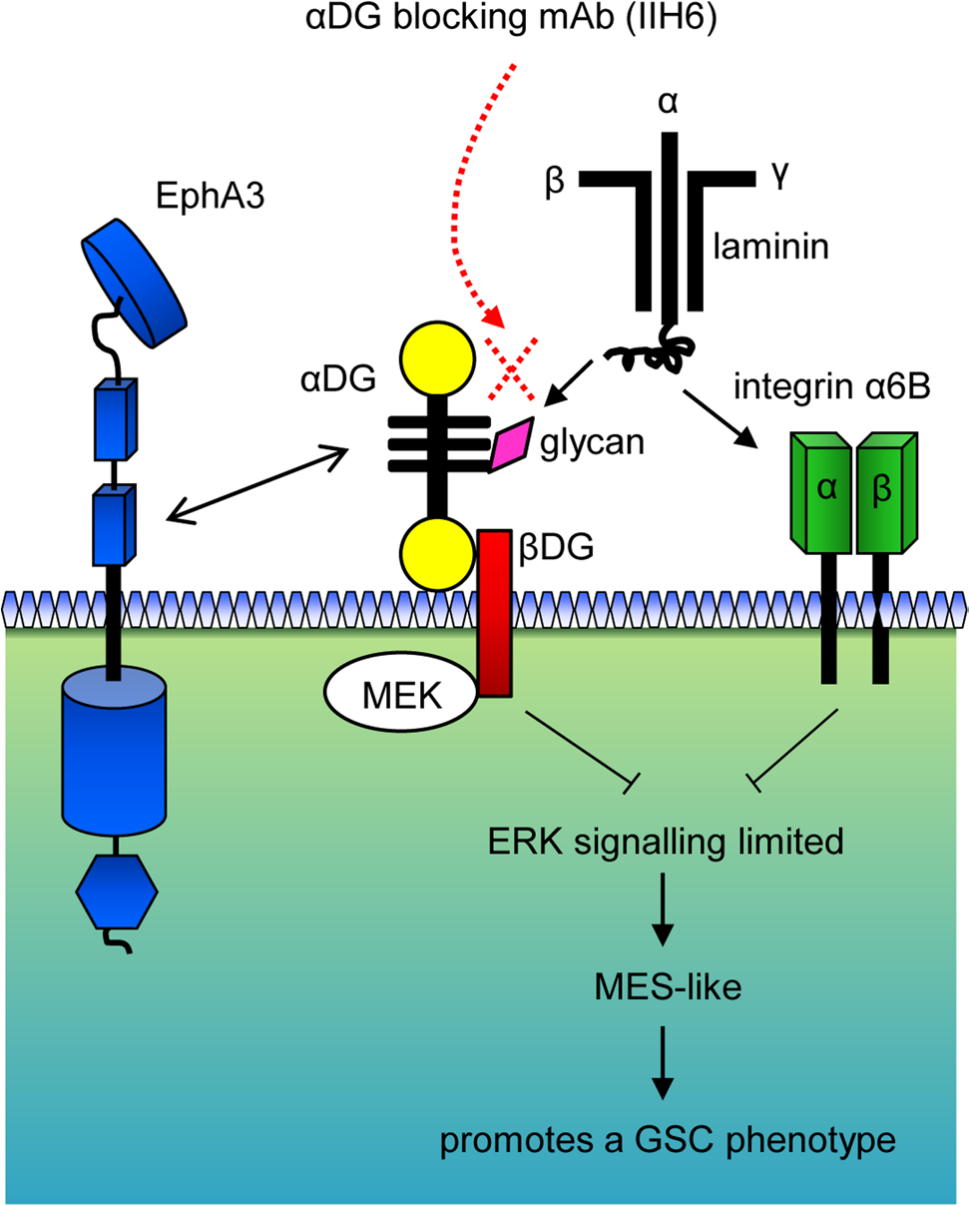
The dystroglycan complex supresses MAPK activation to regulate glioma Stem cell commitment. DG, when bound to laminin, cooperates with EphA3 and integrin α6B in the vascular niche to maintain a GSC mesenchymal phenotype. Functionally, DG and integrin α6B limit sustained ERK activation preventing GSC differentiation. Blockade of αDG glycosylation sites, using the IIH6 mAb, induced sustained ERK1/2 activation, leading to translocation of ERKs to the nucleus followed by GSC differentiation and reduced GBM aggressiveness

While the integrin-α6A splice variant, through interaction with laminin, can activate the ERK pathway, our mRNA expression analysis of 28 tumour specimens showed that the integrin-α6B splice variant was predominantly expressed in GBM and correlated with mesenchymal markers. α6B is strongly linked with a mesenchymal stem cell signature in breast cancer [26], and has a truncated intracellular domain which cannot actively signal the ERK pathway [61]. Thus, in GBM when αDG is bound to BM proteins such as laminin, βDG most likely sequesters MEK and ERK to prevent ERK pathway activation, [23, 56] (see model Fig. 7). Our findings indicate that the α-subunit of the DG receptor, when bound to laminin, is predominantly acting as a brake on ERK activation, thereby maintaining GSCs and the MES-like population within BM-rich regions and niches within these tumours.

DG is expressed widely throughout the body, especially in skeletal muscle, making potential therapeutic targeting of this receptor challenging. It appears that unique brain dystroglycan glycoepitopes have been identified and antibodies raised to recognise these unique glycan moieties [40]. It is conceivable that an αDG brain-specific targeting antibody could be developed, though targeting of normal brain would still be undesirable. This could be overcome through the use of bi-specific targeting strategies with tumour-specific antigens, such as EphA3.

Reduction or loss of DG expression has been reported in several epithelial tumours such as breast, colon, and prostate cancers, implicating the receptor in tumour invasion and dissemination [30, 55]. A potential mechanism to explain this phenomenon is degradation of βDG by MMPs in the TME [33]. Additionally, defects in αDG glycosylation may also play a role in cancer progression. A number of cancers of epithelial origin show an association between loss of αDG glycosylation and tumour progression. A report of this mechanism has also been shown for GBM [1]. Among several αDG glycosylation related genes, *LARGE* may have a great impact on cancer biology and the silencing of *LARGE* promotes cancer cell migration and anchorage-dependent growth [15, 21]. Contrary to these studies, our findings indicate that glycosylated αDG maintains a robust MES-like phenotype likely by promoting interaction with BM proteins in the TME and further acts to anchor GSCs within the vascular niche. Given the significant heterogeneity present in GBM, both mechanisms are likely true, where αDG glycosylation is tuned to maintain MES-like or GSC states, as tumour cells exit these niches, αDG expression is lost and tumour cell differentiation and state change occurs.

## Electronic supplementary material

Below is the link to the electronic supplementary material.

Supplementary material 1 (DOCX 4808 kb)

## Data Availability

Primary GBM cell line characterisation data is publicly available from Q-Cell QIMR Berghofer https://www.qimrberghofer.edu.au/q-cell/.
