# Supplementary material for: The dystroglycan receptor maintains glioma stem cells in the vascular niche
Source: Acta Neuropathol. 2019 Aug 28;138(6):1033–52. doi: 10.1007/s00401-019-02069-x (PMC6851226; doi:10.1007/s00401-019-02069-x)
Supplement: Supplementary file 1 — Supplementary material 1 (DOCX 4808 kb) [file 401_2019_2069_MOESM1_ESM.docx]

**Online Resources**

**The Dystroglycan Receptor Maintains Glioma Stem Cells in the Vascular Niche**

Bryan W. Day,^1,2,3*^ Justin D. Lathia,^4^ Zara C. Bruce,^1^ Rochelle C.J. D’Souza,^1^ Ulrich Baumgartner,^1^ Kathleen S. Ensbey,^1^ Yi Chieh Lim,^1^ Brett W. Stringer,^1^ Seçkin Akgul,^1^ Carolin Offenhäuser,^1^ Yuchen Li,^1^ Paul R. Jamieson,^1^ Fiona M. Smith,^1^ Courtney L.R. Jurd,^1^ Thomas Robertson,^5^ Po-Ling Inglis,^5^ Zarnie Lwin,^5^ Rosalind Jeffree,^5^ Terrance G. Johns,^6^ Krishna P.L. Bhat,^7^ Jeremy N. Rich,^8^ Kevin P. Campbell,^9^ and Andrew W. Boyd^1,3^

^1^Department of Cell and Molecular Biology, QIMR Berghofer Medical Research Institute, Brisbane, Queensland, 4006, Australia.

^2^School of Biomedical Sciences, Faculty of Health, Queensland University of Technology, Brisbane 4059, Australia

^3^School of Biomedical Sciences, The University of Queensland, Brisbane 4072, Australia

^4^Cleveland Clinic, Lerner College of Medicine, Case Western Reserve University, Cleveland, Ohio, OH 44195, United States.

^5^Royal Brisbane and Women’s Hospital, Brisbane, Queensland, 4006, Australia.

^6^Telethon Kids Institute, Perth, Western Australia, 6009, Australia.

^7^MD Anderson Cancer Center, The University of Texas, Houston, Texas, TX 77030, United States.

^8^Medicine Department, University of California, San Diego, La Jolla, California, CA 92093-0021, United States.

^9^Department of Molecular Physiology and Biophysics, and Department of Neurology, Roy J. and Lucille A. Carver College of Medicine, Howard Hughes Medical Institute, University of Iowa, Iowa City, IA 52242, United States.

^*^ Lead Contact: Prof Bryan W Day – Group Leader, Sid Faithfull Brain Cancer Laboratory, QIMR Berghofer MRI. +61 4 02 667 3885, [Bryan.Day@qimrberghofer.edu.au](mailto:Bryan.Day@qimrberghofer.edu.au)

**
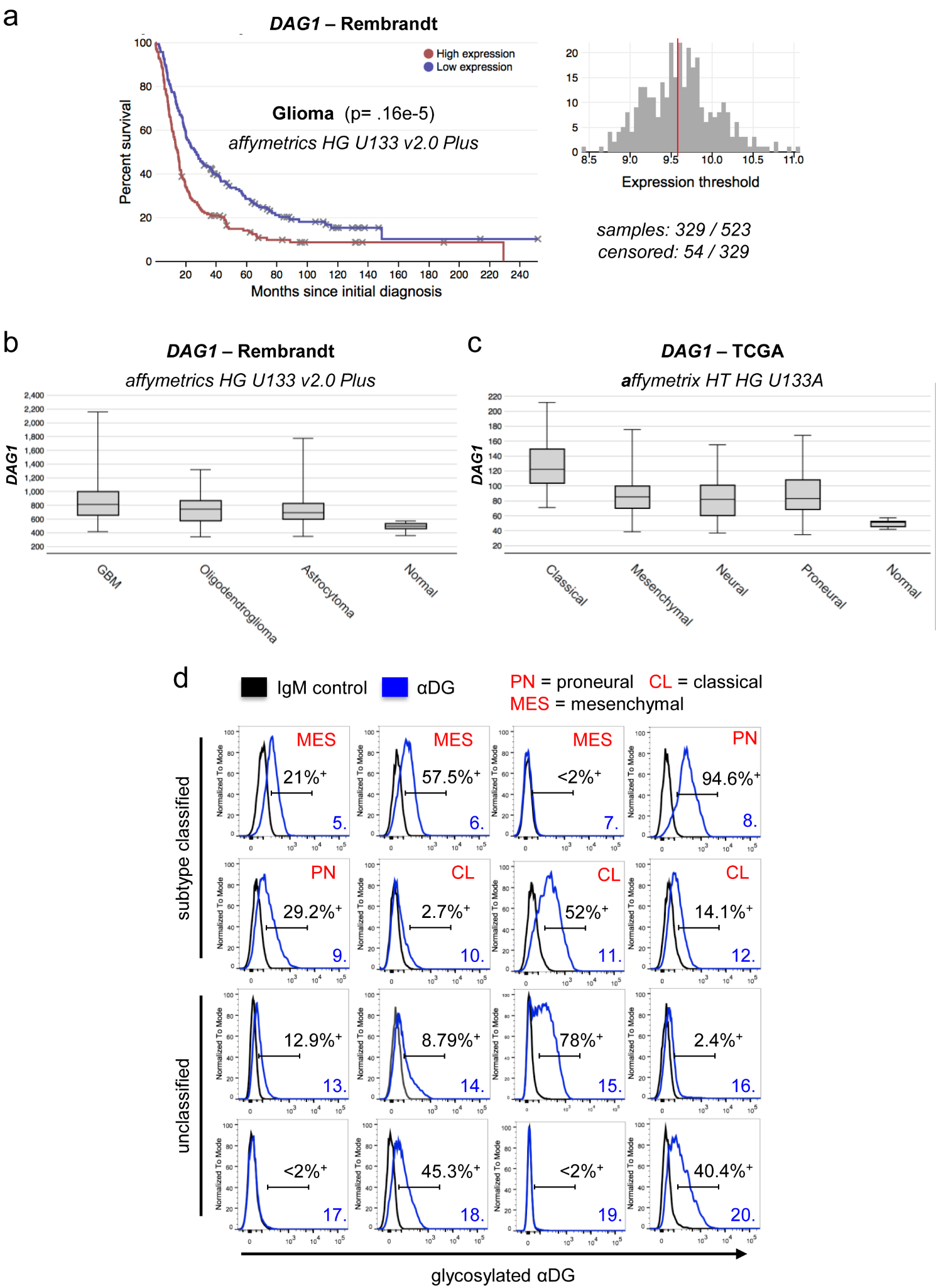
**

**Supplementary Fig. 1** Dystroglycan Correlates with Glioma Patient Outcome and αDG is Abundantly Glycosylated in GBM. **a** *DAG1* expression was correlated to Glioma patient survival (n=523) using the Rembrandt database. **b** *DAG1* expression was compared in a variety of brain tumour types (n=454) to non-tumour brain tissue (n=28) using the Rembrandt database. **c** *DAG1* expression was compared to GBM molecular subtype and non-tumour brain tissue using the TCGA database. **d** Flow cytometric analysis for αDG glycosylation (IIH6 mAb) was performed on early passage primary GBM cell lines grown as serum-free glioma neural stem cell cultures, compared to isotype control. GBM subtypes: (MES = mesenchymal, PN = proneural and CL= classical).

**
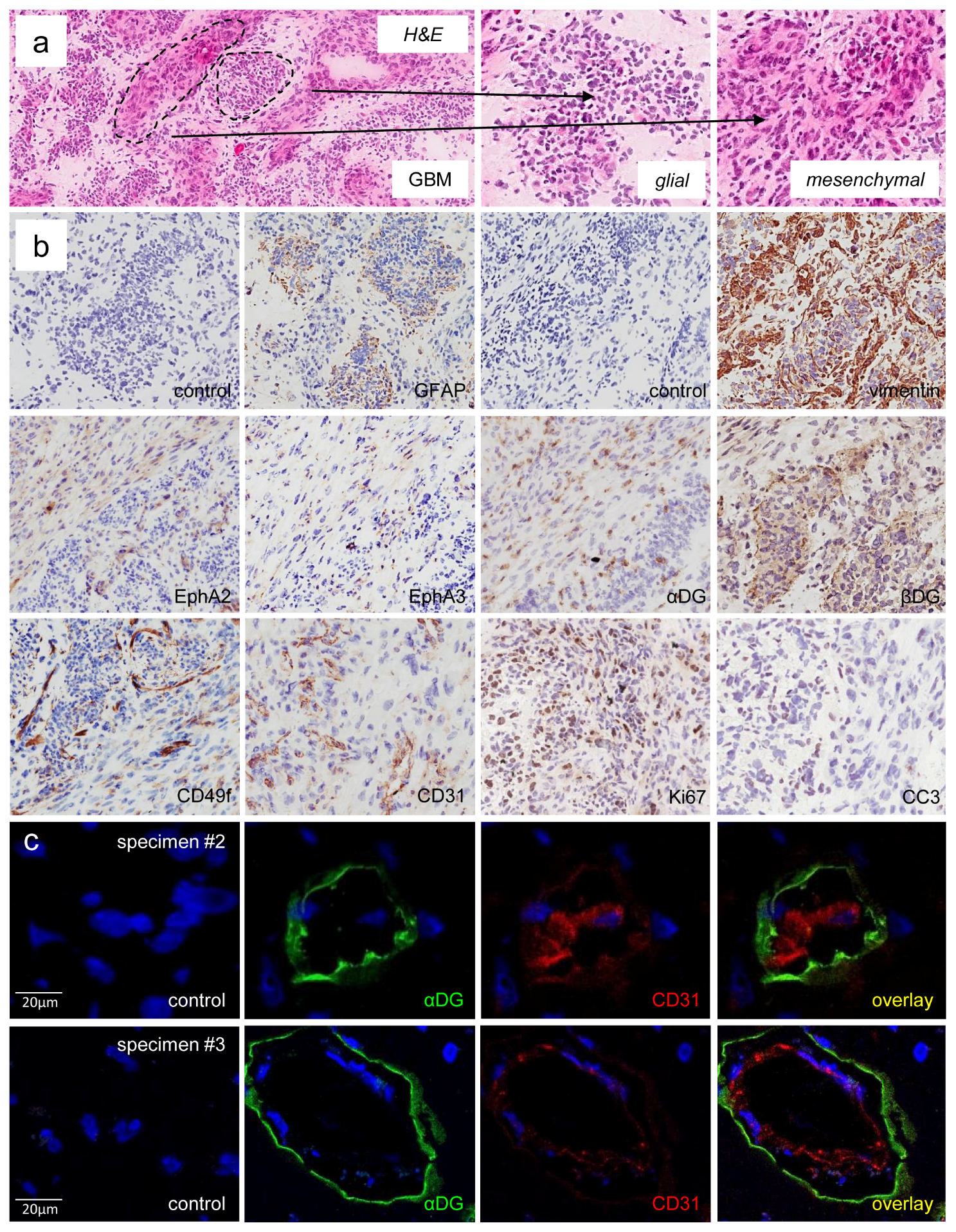
**

**Supplementary Fig. 2** Glycosylated αDG is Expressed in the Vascular Niche and Discretely on Mesenchymal-like Glioma Tissue. **a** H&E section of a GBM patient specimen displaying distinct morphological tumour histology similar to the gliomatous (glial-like) and sarcomatous (mesenchymal-like) tumour elements observed in GS specimens **b** IHC analysis of sequential GBM tissue sections for GFAP, vimentin, EphA2, EphA3, αDG, βDG, CD49f, CD31, Ki67 and cleaved caspase 3 (CC3) and GFAP. **c** IF dual staining of a GBM specimen showing localisation of αDG expression (IIH6 - green) surrounding (CD31^+^ - red) tumour blood vessels.


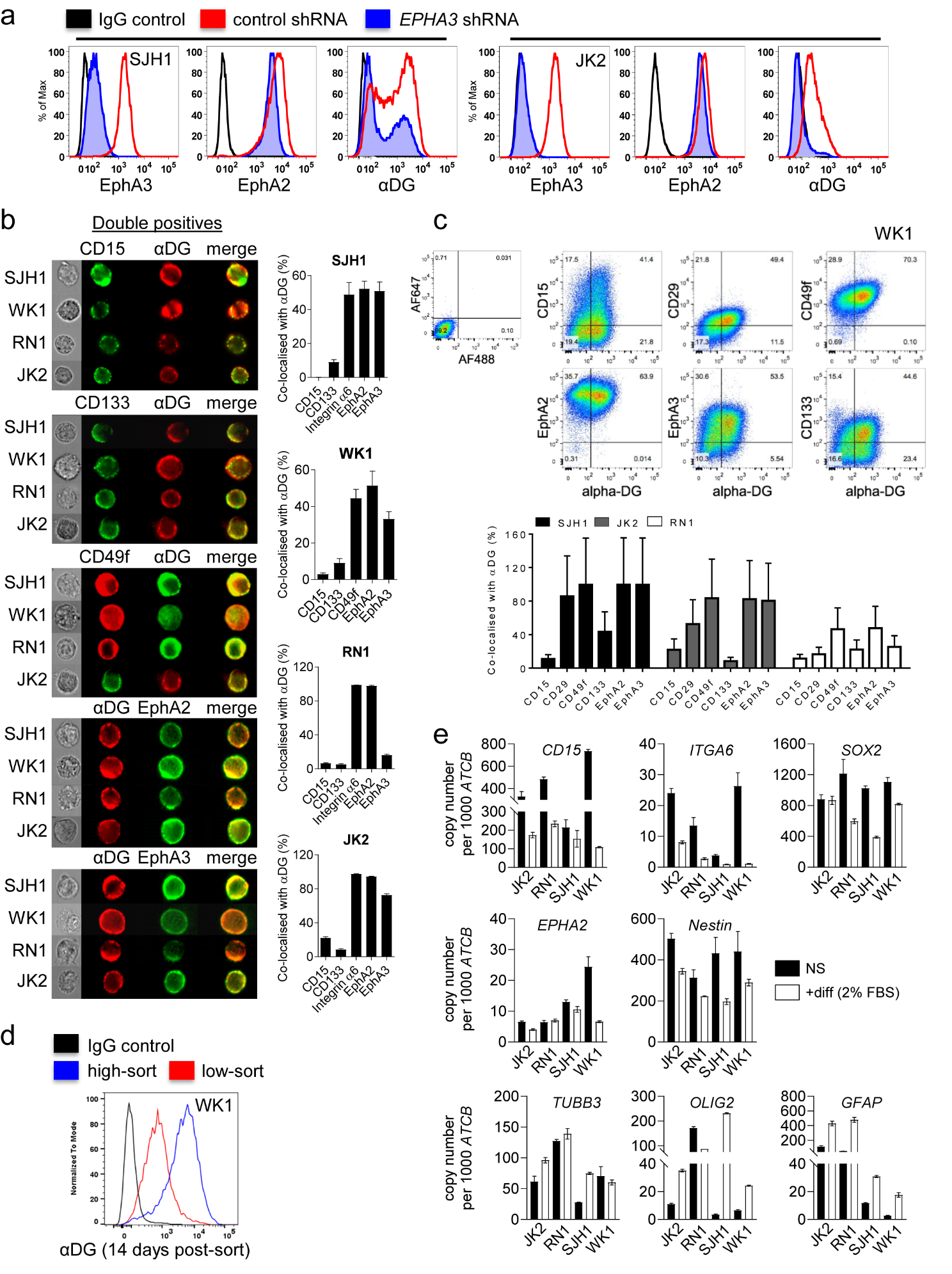


**Supplementary Fig. 3** αDG Interacts with EphA2 and EphA3 Receptors and is Expressed on GSCs. **a** *EPHA3* shRNA mediated KD was performed on two primary GBM cultures (SJH1 and JK2). Following stable cell selection (Neomycin), EphA2 (1F7 mAb), EphA3 (IIIA4) and αDG (IIH6 mAb) protein levels were assessed by flow cytometry in *EPHA3* shRNA (blue) compared to control shRNA (red) expressing cells **b** Amnis flow cytometric analysis was performed on four primary GBM cultures to assess membrane localization of glycosylated αDG, with known GSC markers (CD15, CD133, CD49f, EphA2 and EphA3). **c** Multi-colour flow cytometric analysis was performed on four primary GBM cultures to assess co-expression of glycosylated αDG, with known GSC markers (CD15, CD29, CD133, CD49f, EphA2 and EphA3). **c** αDG^high^ versus αDG^low^ populations were isolated from WK1 cells using FACS, the isolated populations were recombined and αDG expression assessed by flow cytometry 14 days post-sort. **e** GBM neuropshere differentiation was induced using 2% FBS, 3 days post differentiation GSC marker and differentiation marker (*TUBB3, OLIG2* and *GFAP*) expression assessed by QPCR and compared to undifferentiated cells. All data presented as the mean ±SD of three independent experiments.


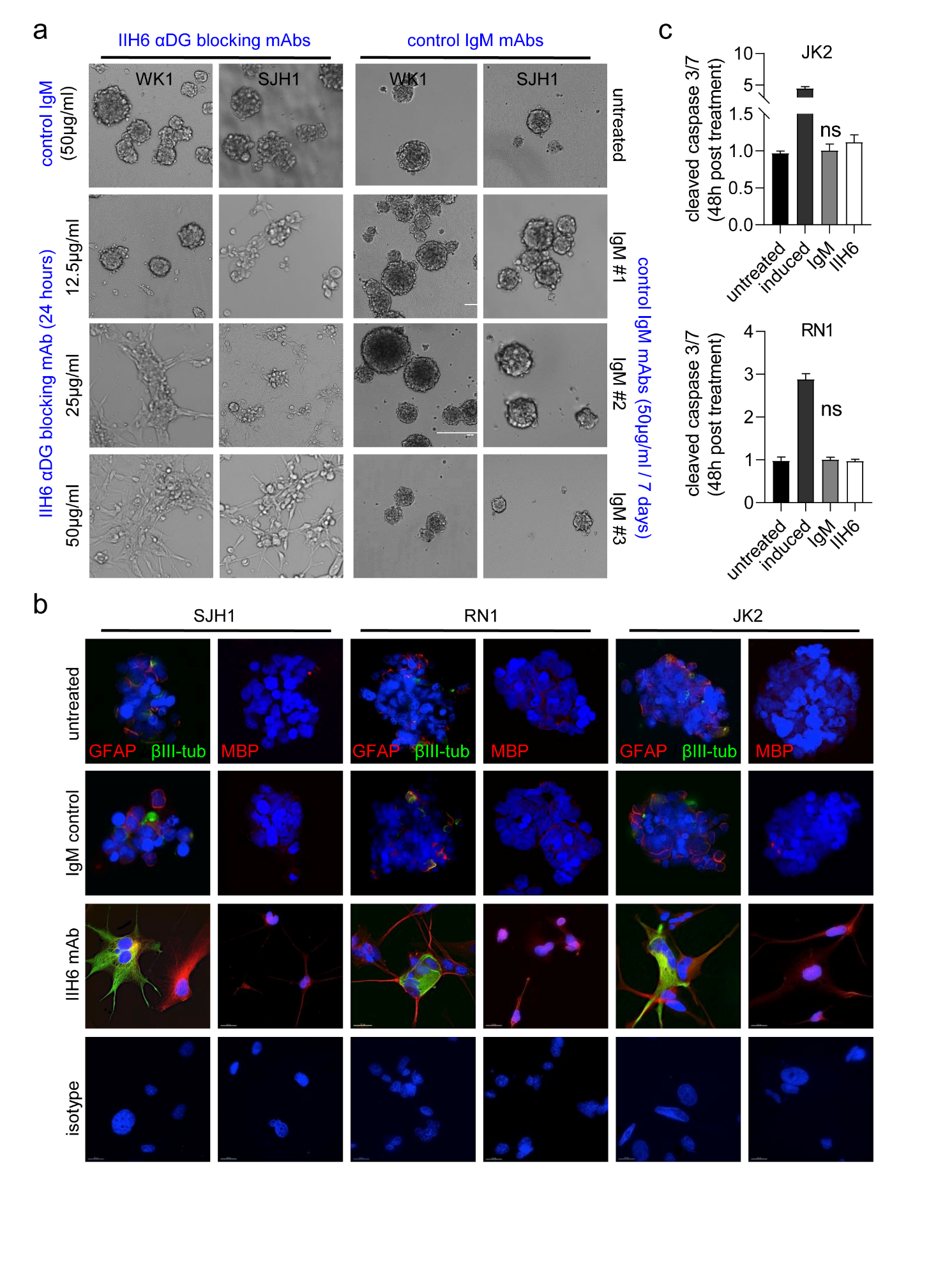


**Supplementary Fig. 4** αDG Blockade Induces GSC Differentiation. **a** An IIH6 dose response (12.5, 25, 50µg/ml) was conducted using WK1 and SJH1 GBM neurospheres compared to an equivalent IgM control (50µg/ml). Bright field images showing neurosphere loss using IIH6 (25 and 50µg/ml) but not IIH6 (12.5µg/ml). Bright field images showing no loss of neuropshere using three independent control IgM mAbs (50µg/ml) **b** Four primary GBM neurosphere cultures treated with the αDG glycosylation blocking mAb (IIH6, 50µg/ml) and an equivalent IgM control (50µg/ml). IF staining was performed 48 hours post IIH6 mAb treatment for the differentiation markers (GFAP-red, βIII-tubulin – green, myelin basic protein (MBP) – red and the nuclear counter-stain DAPI-blue). **c** ApoTox-Glo Triplex assay was used to assess caspase3/7 activity and cell viability 48 hours post IIH6 treatment in primary GBM neurosphere cultures.

**
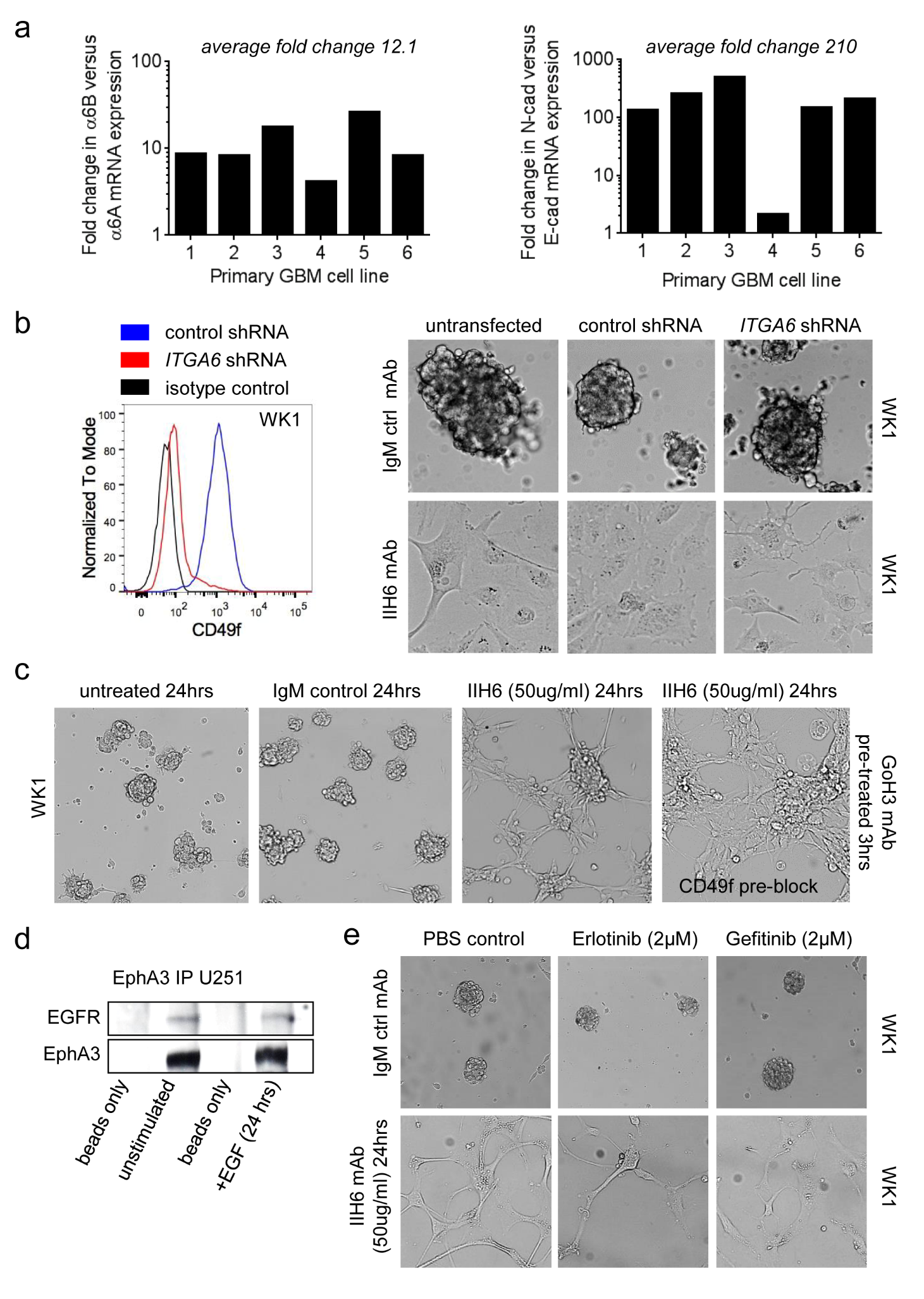
**

**Supplementary Fig. 5** αDG Controls ERK Signalling to Regulate GSCs and Promote MES-like GBM state. **a** Fold change in mRNA expression between (integrin α6A and integrin α6B) and (N-cadherin and E-cadherin), in 6 primary early passage GBM GNS cultures. **b** Flow cytometric analysis of CD49f protein expression following *ITGA6* shRNA mediated KD in WK1 cells. Bright field images of *ITGA6* shRNA versus control shRNA and untransfected WK1 neurospheres 24 hours post treatment with IIH6 (50µg/ml) or an equivalent control IgM control mAb (50µg/ml). **c** Bright field images of WK1 neurospheres pre-treated with GoH3 (10µg/ml) for three hours prior to treatment with IIH6 (50µg/ml) for 24 hours. **d** Immunoprecipitation (IP) for EphA3 was performed from 1mg of lysate from U251-MG GBM cells to compare membrane association of EphA3 with EGFR. Prior to IP, cells were either unstimulated or stimulated with EGF (1ug/ml) for 24 hours. Protein G only was used as a control **e** Bright field images of WK1 neurospheres pre-treated with the EGFR inhibitor Erlotinib (2µM) or Gefitinib (2µM) for three hours prior to treatment with IIH6 (50µg/ml) or an equivalent control IgM control mAb (50µg/ml) for 24 hours.

**
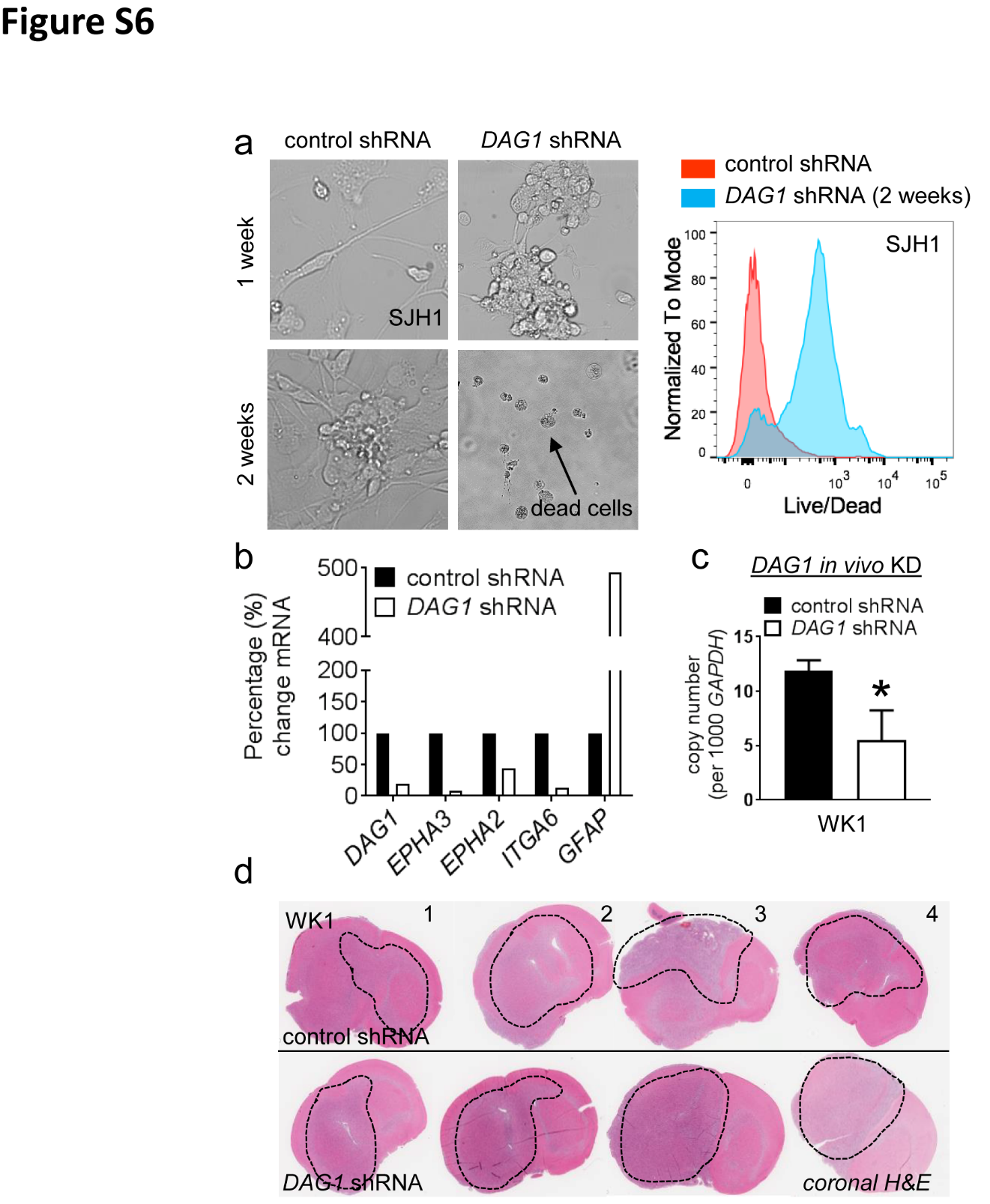
**

**Supplementary Fig. 6** *DAG1* Down Regulation Delays or Prevents GBM Formation *In-Vivo.* **a** Bright field images and propidium iodide (PI) flow cytometric analysis of SJH1 neurospheres 2 weeks post *DAG1* shRNA KD compared to control shRNA transfected cells. **b** QPCR analysis showing the percentage change in mRNA expression levels between of *DAG1, EPHA2, EPHA3, ITGA6 and GFAP* in JK2 *DAG1* shRNA KD versus control shRNA transfected cells. **c** QPCR analysis confirming a significant (*p<0.01) reduction in *DAG1* mRNA levels compared to control shRNA WK1 engrafted animals (n=4 from each group was analysed). **d** Representative H&E coronal sections from each WK1 *DAG1* shRNA versus control shRNA engrafted animal following euthanasia from either illness or tumour burden.
